# PAFAH1B3 Exists in Linear Chromosomal and Extrachromosomal Circular DNA and Promotes HCC Progression via EMT

**DOI:** 10.3390/ijms26188801

**Published:** 2025-09-10

**Authors:** Dandan Li, Huishan Sun, Yingjie Wang, Yicong Yin, Ying Zhu, Xia Qian, Shanshan Wang, Longhao Zhang, Haitao Zhao, Ling Qiu

**Affiliations:** 1 Department of Laboratory Medicine, State Key Laboratory of Complex Severe and Rare Diseases, Peking Union Medical College Hospital, Chinese Academy of Medical Science and Peking Union Medical College (CAMS & PUMC), Beijing 100730, China; lidandan@pumch.cn (D.L.); yicongyin@foxmail.com (Y.Y.); zhuyingcpu@163.com (Y.Z.); qx286331722@gmail.com (X.Q.); 2 Center for Biomarker Discovery and Validation, National Infrastructures for Translational Medicine, Institute of Clinical Medicine, Peking Union Medical College Hospital, Chinese Academy of Medical Science and Peking Union Medical College (CAMS & PUMC), Beijing 100730, China; sunhuishan1990@163.com; 3 Department of Orthopedic Surgery, State Key Laboratory of Complex Severe and Rare Diseases, Peking Union Medical College Hospital, Chinese Academy of Medical Science and Peking Union Medical College (CAMS & PUMC), Beijing 100730, China; 15813653746@163.com; 4 Department of Liver Surgery, State Key Laboratory of Complex Severe and Rare Diseases, Peking Union Medical College Hospital, Chinese Academy of Medical Sciences and Peking Union Medical College (CAMS & PUMC), Beijing 100730, China; shanwangwhu@163.com (S.W.); zlonghao16@gmail.com (L.Z.)

**Keywords:** eccDNAs, PAFAH1B3, hepatocellular carcinoma, epithelial-to-mesenchymal transition

## Abstract

Recent evidence highlights the role of extrachromosomal circular DNAs (eccDNAs) in cancers. However, reports regarding its role in hepatocellular carcinoma (HCC) are infrequent. The abundance of eccDNAs from five HCC/adjacent tissue pairs was explored using Circle-Sequencing. eccDNA PAFAH1B3 was selected as one of the objects. The effect of eccDNA PAFAH1B3 on HCC progression was determined using EdU, Transwell, and apoptosis assays. Additionally, the expressions of eccDNA PAFAH1B3, mRNA PAFAH1B3, and epithelial–mesenchymal transition (EMT)-related markers were determined using RT-PCR and WB. A xenograft tumor model was established to explore the function of PAFAH1B3 in vivo, and EMT-related markers were detected using RT-PCR and IHC analyses. The abundance of eccDNA PAFAH1B3 was significantly increased in HCC cell lines after transfection with eccDNA PAFAH1B3, and promoted the proliferation, migration, and invasion of liver cells while inhibiting apoptosis. The levels of mRNA PAFAH1B3 were also upregulated. Furthermore, intratumoral injection of PAFAH1B3 inhibitor suppressed tumor growth, and PAFAH1B3 knockdown increased and decreased the levels of the E-cadherin and N-cadherin, respectively. Our study findings reveal that eccDNA PAFAH1B3 may promote the occurrence and development of HCC by enhancing the expression of PAFAH1B3 and regulating EMT.

## 1. Introduction

Extrachromosomal circular DNAs (eccDNAs) are a class of circular DNA molecules that exist independently of chromosomes [1]. These molecules are structurally circular, relatively stable, and resistant to nuclease degradation. They were initially discovered in the cell nucleus in 1964 and have since been found to be widely distributed in animals, plants, fungi, and other organisms [2]. The mechanisms of eccDNA formation include chromosome breakage, DNA damage, non-homologous end joining, stalling, template switching during DNA replication, and formation of R-loops during transcription [3,4]. EccDNAs are mapped to over one hundred thousand unique loci in the genome and are enriched in specific regions, including transcriptionally active chromatin [5,6]. They play important roles in genetic variation, evolution, genomic instability and plasticity, drug resistance, environmental adaptation, mutation, and tumor development [1,7,8].

EccDNAs are carriers of oncogene amplification that exhibit higher chromatin accessibility than chromosomal DNA. Transcriptional regulatory factors are likely to bind to these molecules, resulting in higher transcriptional activity [9]. Additionally, they do not follow a Mendelian distribution. Hence, through asymmetric distribution, those carrying oncogenes and drug-resistance genes accumulate in tumor cells rapidly, driving heterogeneity within the tumor [10]. For example, in neuroblastoma, eccDNAs reassemble into linear genomes through fusion circularization, driving the genomic remodeling and expression of oncogenes [11].

Recently, the emergence of high-throughput sequencing technologies and bioinformatic methods has greatly advanced research on eccDNAs in cancer [12,13,14,15]. Previous reports highlight the role of eccDNAs in the development of neuroblastoma, glioblastoma, colon, breast, cervical, and ovarian cancer, among others [16,17,18]. However, studies on the role of eccDNAs in hepatocellular carcinoma (HCC) are limited. Notably, HCC is the second most common cause of cancer-related deaths worldwide, representing about 90% of all primary liver cancer cases [19]. In previous studies, four pairs of liver cancer and adjacent tissues were sequenced, leading to a report regarding the whole-genome landscape of eccDNAs in liver cancer. Furthermore, synthetic eccDNAs containing the miR-17–92 clusters promote cell proliferation, migration, and invasion when transfected into liver cancer cells [20,21]. However, to date, there are no molecular mechanistic studies on how eccDNAs promote or inhibit the development of liver cancer or on the establishment of subsequent treatment systems. Researchers have compared the differentially expressed genes identified using transcriptome sequencing with the protein-coding genes carried by differentially expressed eccDNAs. The findings revealed that mRNA SLC16A3 and BAIAP2L2, which are highly expressed in liver cancer tissues, are associated with the upregulation of eccDNA SLC16A and BAIAP2L2, respectively. Furthermore, their expression negatively correlates with the survival rate of patients with liver cancer, indicating that eccDNAs can exert their effects by interfering with mRNA transcription [22].

Platelet-activating factor acetylhydrolase 1B3 (PAFAH1B3), a catalytic subunit of PAFAH, is important for apoptosis, metastasis, and angiogenesis in cancer. PAFAH1B3 is involved in diverse cancer-related signaling pathways, including PAF and WNT, and facilitates cancer progression [23]. Moreover, evidence suggests that PAFAH1B3 is upregulated in liver cancer cell lines, and knockdown of this gene significantly inhibits cell proliferation, migration, and invasion in HCC. In addition, it has been correlated with poor prognosis in non-small cell lung cancer (NSCLC) and HCC [24]. In the preliminary stage of this research project, we sequenced five pairs of liver cancer and adjacent tissues. We annotated the differentially expressed eccDNAs and intersected them with transcriptome data from the TCGA database (https://portal.gdc.cancer.gov, accessed on 15 May 2023). Subsequently, we searched the GEPIA2 database (http://gepia2.cancer-pku.cn/, accessed on 17 May 2023) and conducted in vitro functional experiments to identify eccDNA PAFAH1B3 as one of the objects of this study. Notably, after transfection with eccDNA PAFAH1B3 in the cell lines, the levels of mRNA PAFAH1B3 were also upregulated. The abundance of eccDNA PAFAH1B3 in cells is relatively low, and there is currently no well-established method for knocking out genes on eccDNA. This study silenced the mRNA of PAFAH1B3 to investigate its role in cancer. Reportedly, elevated expression of PAFAH1B3 affects EMT, thereby promoting papillary thyroid carcinoma [25] and lung adenocarcinoma [26] cell proliferation and metastasis. However, currently, no reported evidence exists on whether PAFAH1B3 plays a role in HCC through the process of EMT.

This study aims to investigate the role of eccPAFAH1B3 in the progression of HCC and to examine whether si-PAFAH1B3 acts as an inhibitor of HCC progression through the EMT process. The findings may offer a novel research avenue for targeted HCC therapy.

## 2. Results

### 2.1. Expression Profiles of eccDNAs in Liver Cancer and Adjacent Non-Cancerous Tissues

**Figure 1:**
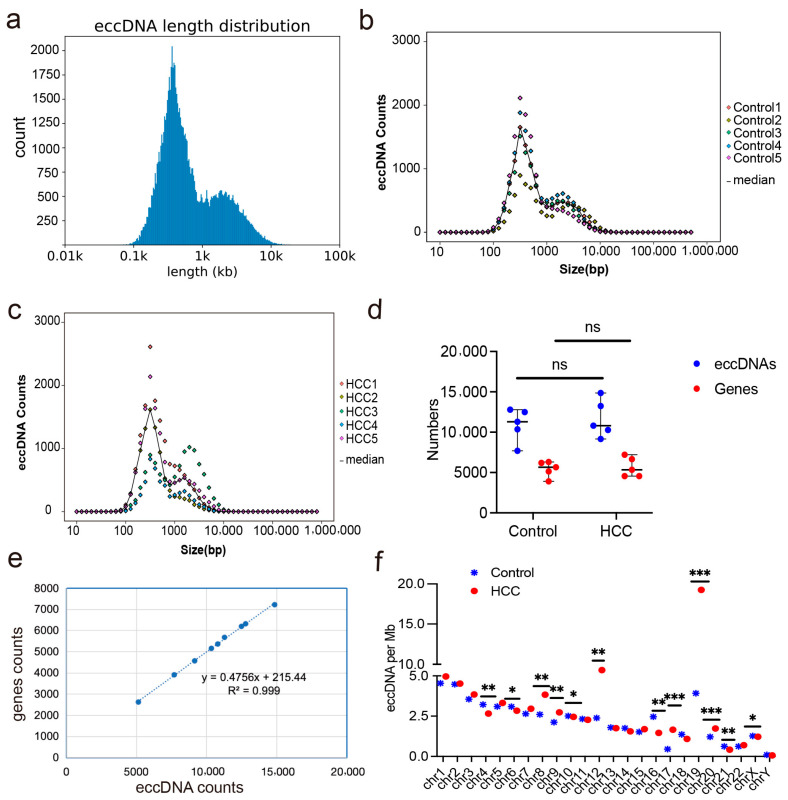
The abundance of eccDNAs in liver cancer and adjacent non-cancerous tissues. (**a**) Length distribution of eccDNAs sequenced in five liver cancer tissues and adjacent non-cancerous tissues. (**b**) Length distribution of eccDNAs sequenced in five adjacent non-cancerous tissues. (**c**) Length distribution of eccDNAs sequenced in five liver cancer tissues. (**d**) The number of eccDNAs and eccDNAs mapped to protein-coding genes in tissue sequencing data. (**e**) Correlation between the number of eccDNAs and the number of protein-coding genes in tissue sequencing data. (**f**) Differences in the number of eccDNAs per megabase pairs produced by different chromosomes. * *p* < 0.05, ** *p* < 0.01, *** *p* < 0.001, ns: no statistical difference; HCC: hepatocellular carcinoma.

In total, 91,971 eccDNAs (49,607 in liver cancer tissues and 42,364 in adjacent non-cancerous tissues) were identified using Circle-seq of five liver cancer tissues and adjacent non-cancerous tissues. The lengths of the eccDNAs ranged from 35 to 9,868,462 bp (35–9,868,462 bp in liver cancer tissues, and 56–3,920,856 bp in adjacent non-cancerous tissues), with a median value of 490 bp (Figure 1a). The majority of the eccDNAs had lengths between 0.1 and 10 kb (99.90% in liver cancer tissues and 99.60% in adjacent noncancerous tissues), and the length peaks of both groups were between 346 and 378 bp (Figure 1b,c). The number of eccDNAs in liver cancer tissues (*n* = 49,607) was approximately 1.17-fold higher than that in adjacent non-cancerous tissues (*n* = 42,364), indicating an increased genomic instability in liver cancer tissues.

Based on gene annotation analysis, 45,564 eccDNAs (49.54%) were mapped to protein-coding gene regions, with 24,513 and 21,051 eccDNAs in liver cancer tissues and adjacent non-cancerous tissues, respectively, demonstrating that the two groups did not differ significantly (Figure 1d). Additionally, the number of eccDNAs per megabase pair correlated positively with the number of protein-coding genes per megabase pair (*p* < 0.001) (Figure 1e). Chromosomes rich in genes contribute more eccDNA per megabase pair, such as chromosome 19, which has a high gene content (Figure 1f). We mapped the identified 91,971 eccDNAs to genomic regions, including 5′ UTR (untranslated region), exons, CpG islands, and Alu regions to further understand the distribution of eccDNAs.

### 2.2. Differentially Expressed eccDNAs in Liver Cancer and Adjacent Non-Cancerous Tissues

**Figure 2:**
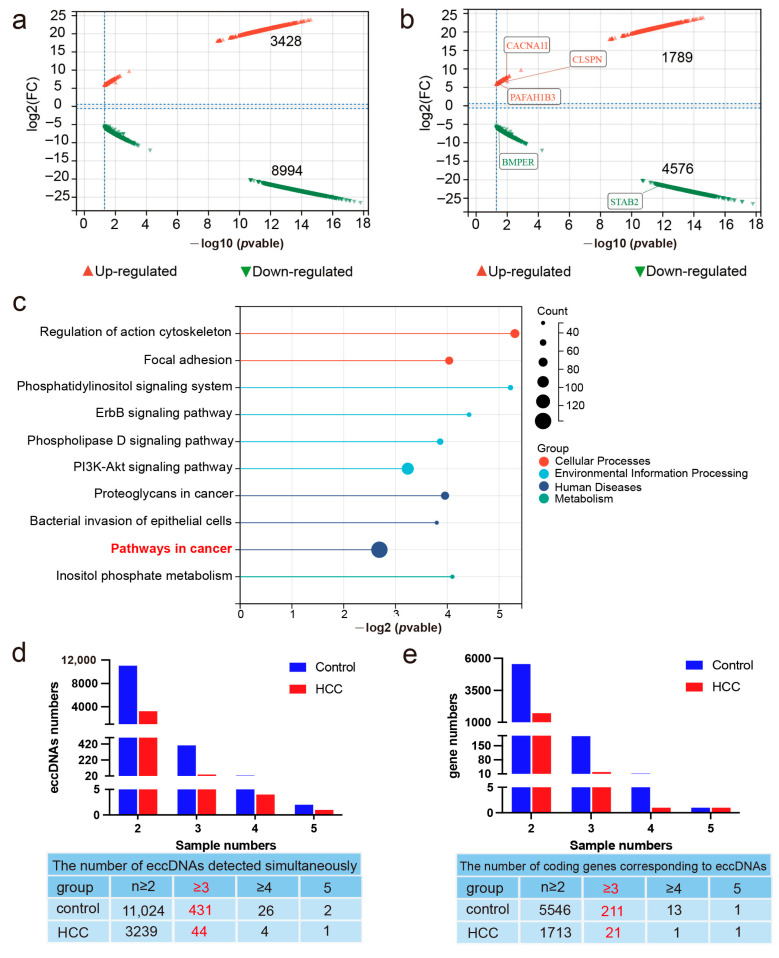
Differentially expressed eccDNAs in liver cancer and adjacent non-cancerous tissues. (**a**) The number of upregulated and downregulated differentially expressed eccDNAs in liver cancer and adjacent non-cancerous tissues. (**b**) The number of upregulated and downregulated differentially expressed eccDNAs mapped to protein-coding genes in liver cancer and adjacent non-cancerous tissues. (**c**) KEGG enrichment analysis of differentially expressed genes. (**d**) The number of eccDNAs detected simultaneously in samples. (**e**) The number of genes corresponding to eccDNAs detected simultaneously in samples.

Differential eccDNA expression in liver cancer and adjacent non-cancerous tissues was detected using Circle-seq. In total, 3428 upregulated and 8994 downregulated eccDNAs were identified (|log2FC| > 1, *p* < 0.05) (Figure 2a). Gene annotation analysis revealed that 52.19% of the upregulated eccDNAs were mapped to protein-coding genes, whereas 50.88% of the downregulated eccDNAs were mapped to protein-coding genes (Figure 2b). KEGG signaling pathway analysis of the differentially expressed genes showed that these genes participated in various pathways, including the ErbB, PI3K-Akt, and phospholipase D signaling pathways, which are involved in regulating cell proliferation, differentiation, metabolism, motility, and migration processes (Figure 2c). Further analysis was performed on differentially expressed eccDNAs detected in at least three pairs of samples. Of these, 44 upregulated and 431 downregulated eccDNAs were detected in at least three pairs of samples (Table S1, of which 21 and 211 were mapped to protein-coding genes (Figure 2d,e, Table S2). Transcriptional sequencing data from the TCGA database were obtained, including 374 liver cancer samples and 50 control samples, where 8739 differentially expressed mRNAs (|log2FC| > 2, *p* < 0.05) were identified, including 8461 upregulated mRNAs and 278 downregulated mRNAs. Furthermore, intersection analysis between the gene annotations of eccDNAs and differentially expressed genes was performed, followed by a search of the GEPIA2 database to explore the potential roles of these genes in liver cancer. Among the genes carried by the three eccDNAs PAFAH1B3, CACNA1, CLSPN (Figure S1), PAFAH1B3 showed the highest expression levels and was correlated with lower patient survival rates (*p* < 0.05). Therefore, in this study, the eccDNA PAFAH1B3 (chr19:42803212-42803548) was selected as one of the objects for further investigation.

### 2.3. Expression Quantification and Circularity Verification of eccDNA PAFAH1B3 in Tissues and Cell Lines

**Figure 3:**
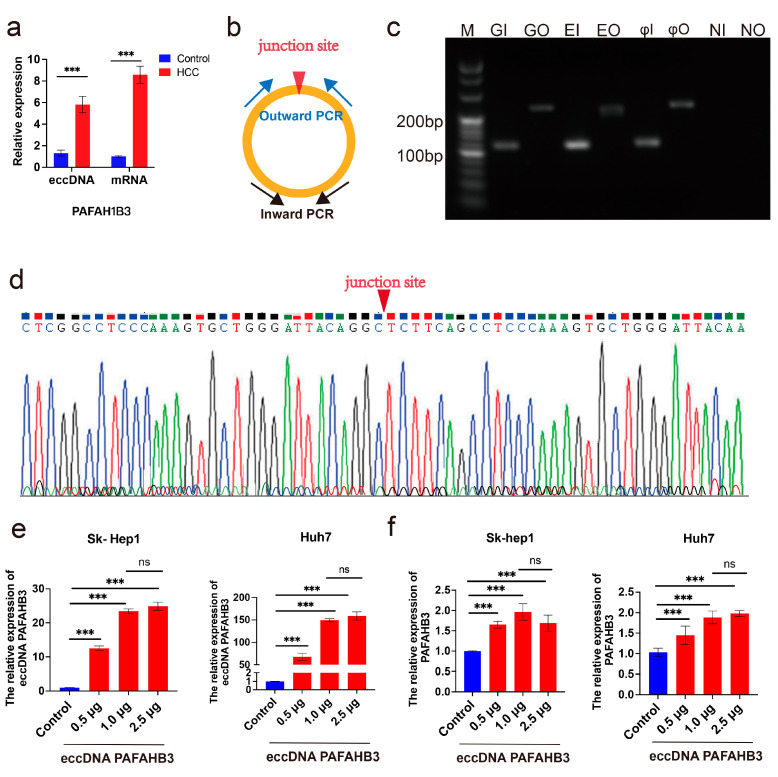
Expression quantification and circularity verification of eccDNA PAFAH1B3 in tissues and cell lines. (**a**) Expression levels of eccDNA PAFAH1B3 and mRNA PAFAH1B3 in liver cancer and adjacent non-cancerous tissues. (**b**) Schematic diagram of eccDNA PAFAH1B3 and primer design for inward and outward PCR. (**c**) Gel electrophoresis of inward and outward PCR products of eccDNA PAFAH1B3. (**d**) Sanger sequencing to determine the circular junction point sequence, indicated by a red inverted triangle at the junction site. (**e**) Expression levels of eccDNA PAFAH1B3 in Sk-hep1 and Huh7 cells after transfection with different amounts of synthetic eccDNA PAFAH1B3. (**f**) Expression levels of PAFAH1B3 in Sk-hep1 and Huh7 cells after transfection with different amounts of synthetic eccDNA PAFAH1B3. *** *p* < 0.001, ns: no statistical difference. GI: Genomic DNA Inward PCR; GO: Genomic DNA Outward PCR; EI: Enriched eccDNA Inward PCR; EO: Enriched eccDNA Outward PCR; φI: Phi29 (φ) rolling-circle amplified eccDNA Inward PCR; φO: Phi29 (φ) rolling-circle amplified eccDNA Outward PCR. The sizes of the inward and outward PCR fragments are 115 bp and 261 bp, respectively.

Total DNA was extracted from five liver cancer tissue samples, rolling-circle amplification was performed, and the linear DNA was removed. The expression levels of eccDNA PAFAH1B3 in tissues were detected using qPCR. The results showed that eccDNA PAFAH1B3 was enriched in liver cancer tissues, which is consistent with the sequencing results of this study. The upregulation of mRNA PAFAH1B3 in these tissues (Figure 3a) was also consistent with the bioinformatics analysis results.

To validate the circularity of eccDNA PAFAH1B3, two sets of primers were designed for inward and outward PCR amplification, targeting the junction sequence of eccDNA PAFAH1B3 (Figure 3b). Inward and outward PCR was performed using genomic DNA, enriched eccDNAs, rolling-circle amplified eccDNAs and negative controls. The inward PCR primer did not include the circular junction sequence; however, it generated amplicons from both genomic DNA and eccDNAs. The outward PCR primer included the circular junction sequence and resulted in amplifying abundant eccDNAs in the rolling-circle amplified eccDNA group, confirming the presence of eccDNA PAFAH1B3 (Figure 3c). Sanger sequencing of the qPCR amplicon covering the junction site of eccDNA PAFAH1B3 revealed a circular junction point, GGCTCT, further confirming the circular structure of eccDNA PAFAH1B3 (Figure 3d).

Transfection of artificially synthesized eccDNA PAFAH1B3 into the liver cancer-associated cell line Sk-Hep1 and liver cancer cell line Huh7 showed a dose-dependent effect on eccDNA PAFAH1B3 expression. Compared with the control group, the eccDNA PAFAH1B3 levels increased approximately 13-fold, 23-fold, and 25-fold in Sk-hep1 cells (1 × 10^6^ cells) transfected with 0.5 μg, 1.0 μg, and 2.5 μg of eccDNA PAFAH1B3, respectively (*p* < 0.001). Similarly, the expression level of mRNA PAFAH1B3 increased by approximately 1.65-fold, 1.96-fold, and 1.69-fold, respectively (*p* < 0.001) (Figure 3e,f). In Huh7 cells (1 × 10^6^ cells), transfection with 0.5 μg, 1.0 μg, and 2.5 μg of eccDNA PAFAH1B3 caused approximately 68-fold, 149-fold, and 159-fold increases in the level of PAFAH1B3, respectively (*p* < 0.001). The expression level of mRNA PAFAH1B3 increased approximately 1.45-fold, 1.89-fold, and 1.97-fold (*p* < 0.001) (Figure 3e,f), with no statistically significant difference between the 1.0 μg and 2.5 μg groups regarding the level of eccDNA PAFAH1B3 and mRNA PAFAH1B3. Therefore, the transfection of 1.0 μg/10^6^ cells of eccDNA PAFAH1B3 was selected for further cellular functional experiments.

### 2.4. The Impact of eccDNA PAFAH1B3 on Liver Cancer Cell Proliferation, Migration, Invasion, and Apoptosis

**Figure 4:**
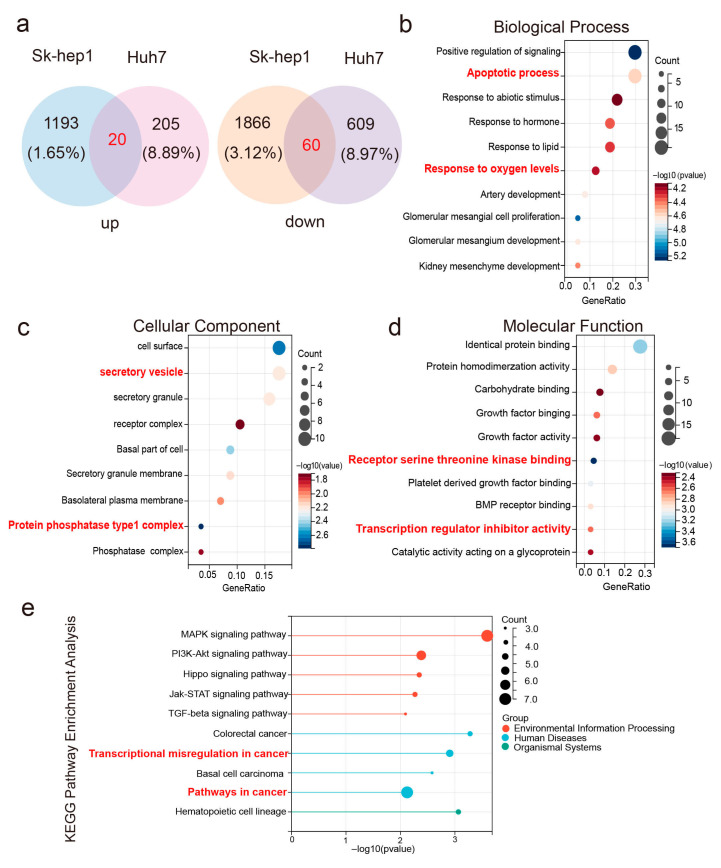
Transcriptome sequencing of liver cancer cells transfected with eccDNA PAFAH1B3: (**a**) differentially expressed genes and their intersections in Sk-hep1 and Huh7 cells transfected with eccDNA PAFAH1B3. (**b**–**d**) GO enrichment analysis of differentially expressed genes: (**b**) biological processes, (**c**) cellular components, (**d**) molecular functions and (**e**) KEGG enrichment analysis of differentially expressed genes. KEGG: Kyoto Encyclopedia of Genes and Genomes; GO: Gene Ontology.

To understand the impact of eccDNA PAFAH1B3 on the phenotype of liver cancer cells, synthetic eccDNA PAFAH1B3 was transfected into Sk-hep1 and Huh7 cells, followed by transcriptome sequencing. The results showed that in both cell lines, 20 and 60 genes were upregulated and downregulated, respectively (|Log2FC| > 1, *p* < 0.05) (Figure 4a). Gene Ontology enrichment analysis revealed that these 80 differentially expressed genes were involved in biological processes, such as cell apoptosis and response to oxygen levels. They also participate in generating vesicle secretion and protein phosphatase complexes and influence mechanisms such as receptor serine–threonine kinase binding and regulation of transcription factor activity (Figure 4b–d). KEGG pathway analysis further unveiled the involvement of these differentially expressed genes in signaling pathways related to cell proliferation, apoptosis, differentiation, and tissue morphogenesis, including MAPK, PI3K-Akt, and Hippo signaling pathways, which play important roles in cancer (Figure 4e).

**Figure 5:**
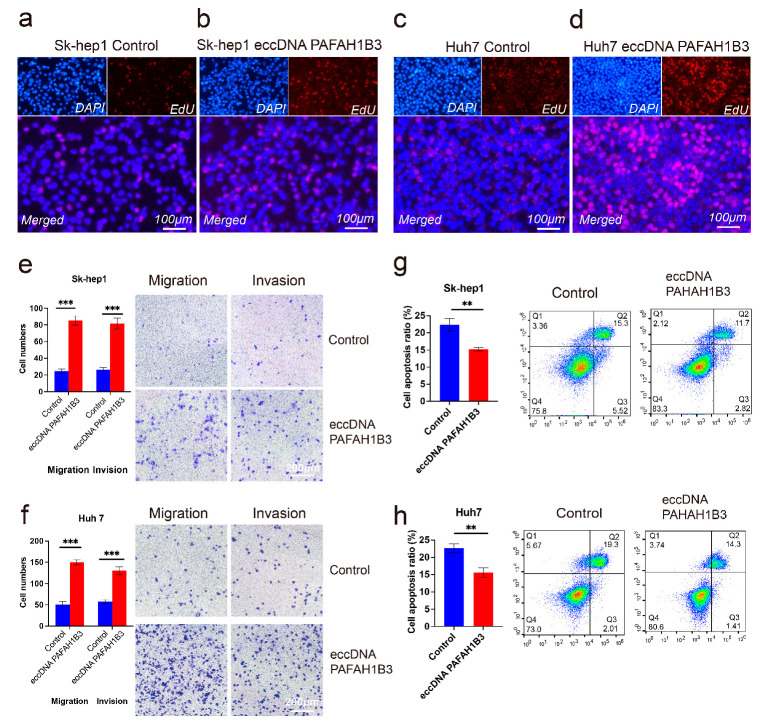
The effect of eccDNA PAFAH1B3 on liver cancer cell proliferation, migration, invasion, and apoptosis. (**a**–**d**) EdU assay to measure cell proliferation: (**a**) Sk-hep1 cells transfected with randomly synthesized DNA circles of equal size as the control group, (**b**) Sk-hep1 cells transfected with synthetic eccDNA PAFAH1B3 as the experimental group, (**c**) Huh7 cells transfected with randomly synthesized DNA circles of equal size as the control group; (**d**) Huh7 cells transfected with synthetic eccDNA PAFAH1B3 as the experimental group. (**e**,**f**) Transwell assay to assess cell migration and invasion abilities of Sk-hep1 and Huh7 cells. (**g**,**h**) Flow cytometry analysis to evaluate apoptosis in Sk-hep1 and Huh7 cells. ** *p* < 0.01, *** *p* < 0.001.

In addition, we investigated the effects of eccDNA PAFAH1B3 on liver cancer cell proliferation, migration, invasion, and apoptosis using EdU, Transwell, and flow cytometry assays. SK-Hep1 and Huh7 cells were transfected with synthetic eccDNA PAFAH1B3. The qPCR results showed that compared with the control group, overexpression of eccDNA PAFAH1B3 promoted the proliferation of SH-Hep1 and Huh7 cells (Figure 5a–d). Furthermore, Transwell experiments revealed that the overexpression of eccDNA PAFAH1B3 significantly enhanced the migration and invasion abilities of liver cancer cells (*p* < 0.001) (Figure 5e,f). Additionally, apoptosis assays demonstrated that the overexpression of eccDNA PAFAH1B3 inhibited apoptosis in SK-Hep1 and Huh7 cells (*p* < 0.05) (Figure 5g,h).

### 2.5. The si-PAFAH1B3 Inhibited the Malignant Progression of HCC In Vitro and In Vivo by Regulating EMT

**Figure 6:**
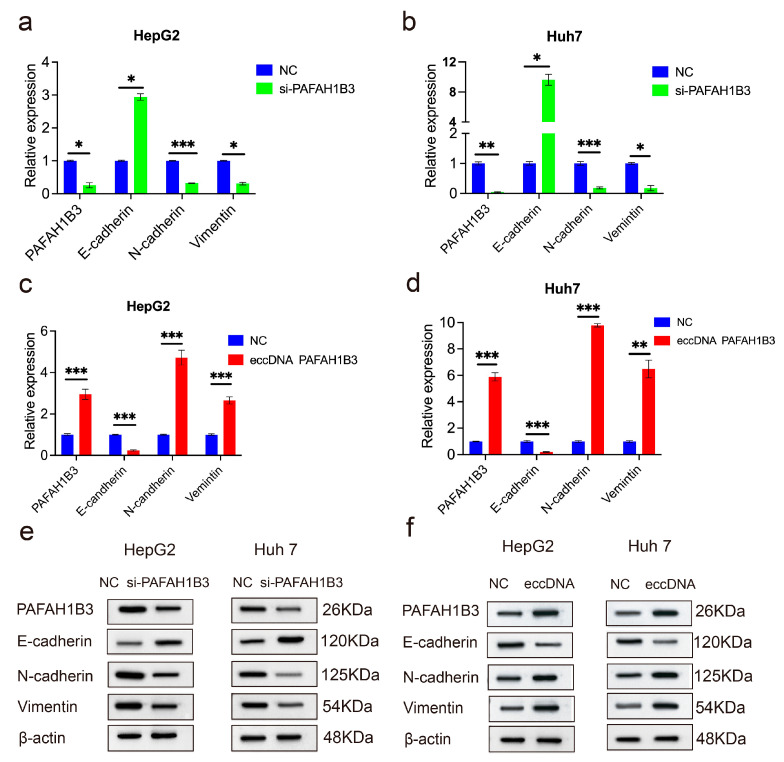
Si-PAFAH1B3 inhibited the malignant progression of HCC by regulating EMT. (**a**,**b**) HepG2 and Huh7 cells were transfected with NC and si-PAFAH1B3; (**c**,**d**) HepG2 and Huh7 cells were transfected with NC and eccDNA PAFAH1B3. (**a**–**d**) PAFAH1B3, E-cadherin, N-cadherin, and Vimentin mRNA were detected through RT-qPCR. (**e**) HepG2 and Huh7 cells were transfected with NC and si-PAFAH1B3; (**f**) HepG2 and Huh7 cells were transfected with NC and eccDNA PAFAH1B3; PAFAH1B3, E-cadherin, N-cadherin, and Vimentin protein expression was detected through Western blotting, respectively. Data are presented as mean ± SD of three independent experiments, * *p* < 0.05, ** *p* < 0.01, *** *p* < 0.001.

**Figure 7:**
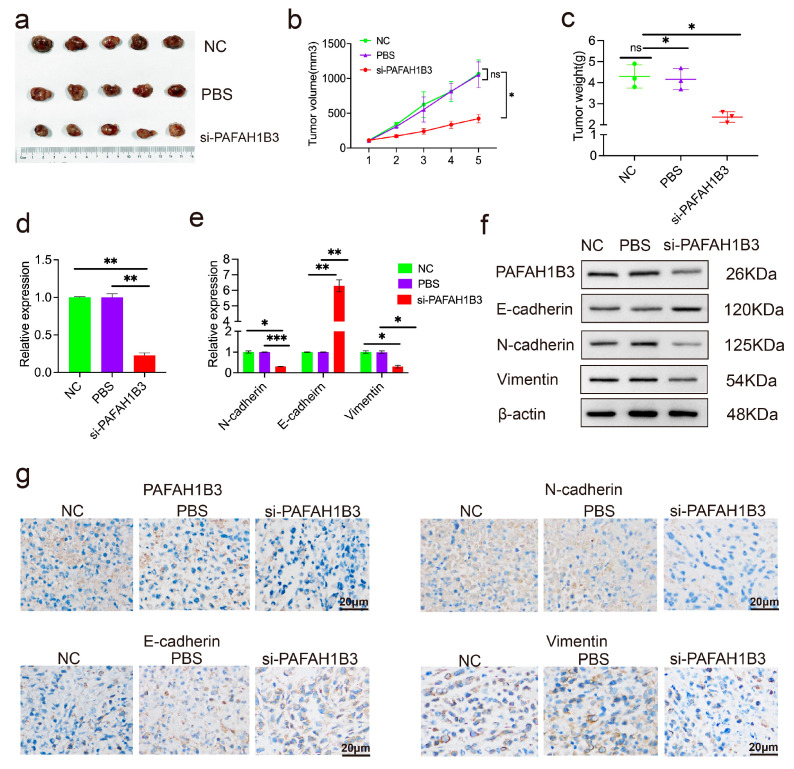
The inhibitory effect of Si-PAFAH1B3 on the growth of HCC tumors in vivo. (**a**) Nude mice were subcutaneously injected with cholesterol-conjugated negative control (NC), phosphate-buffered saline (PBS), and cholesterol-conjugated si-PAFAH1B3. Following five injections, tumors were excised and imaged. (**b**) Tumor volumes were measured prior to injection, and a tumor growth curve was subsequently plotted. (**c**) Tumor weights were recorded on the day of euthanasia. Data are presented as mean ± SD, with n = 5 mice per group. (**d**) The expression levels of PAFAH1B3 in xenograft tumors were quantified using RT-qPCR. (**e**) Expression levels of EMT markers in xenograft tumors were measured using RT-qPCR. (**f**) Expression levels of PAFAH1B3 and EMT markers in xenograft tumors were measured using WB. (**g**) Changes in N-cadherin, E-cadherin, and Vimentin expression in xenograft tumors were detected through IHC staining. Scale bar; 20 μm. * *p* < 0.05; ** *p* < 0.01; *** *p* < 0.001, ns: no statistical difference.

PAFAH1B3 was modulated to determine its effect on EMT. The basal expression levels of PAFAH1B3 in HepG2 and Huh7 cells are shown in Table S3. Of the four PAFAH1B3 siRNAs, si-2 PAFAH1B3 showed the highest silencing efficiency in HepG2 and Huh7 cells (Table S4, Figure S2a,b). Therefore, it was selected for further study. PAFAH1B3 expression decreased after transfection with si-PAFAH1B3 (Figure 6a,b). PAFAH1B3 silencing upregulated the expression of E-cadherin significantly; however, the levels of N-cadherin and Vimentin, which are key biomarkers of EMT, decreased (Figure 6c). The levels of PAFAH1B3, N-cadherin and Vimentin were upregulated, whereas that of E-cadherin was downregulated after transfection with eccDNA-PAFAH1B3 (Figure 6d). We also explored the protein levels of PAFAH1B3 and EMT factors in HepG2 and Huh7 cells after transfection with si-PAFAH1B3 and found that the protein levels of E-cadherin were upregulated; however, the levels of PAFAH1B3, N-cadherin, and Vimentin were downregulated (Figure 6e). Furthermore, the protein levels of PAFAH1B3, N-cadherin, and Vimentin were upregulated, whereas the protein level of E-cadherin was downregulated (Figure 6f). To assess the potential of si-PAFAH1B3 as a therapeutic target, we developed xenograft tumor models by subcutaneously injecting SMMC-7721 cells into nude mice. Following intratumoral administration of cholesterol-conjugated si-PAFAH1B3, a significant reduction in tumor growth and tumor weight was observed compared to the PBS and NC groups (Figure 7a–c). Total RNA was extracted from xenograft tumors, and RT-qPCR was used to measure the expression of PAFAH1B3 and EMT factors. The results showed decreased PAFAH1B3 expression in tumors with PAFAH1B3 silencing (Figure 7d). The expression of N-cadherin and Vimentin was lower than that in the PBS and NC groups after PAHAH1B3 knockdown, whereas E-cadherin expression was higher than that in the PBS and NC groups (Figure 7e). Furthermore, immunohistochemical staining (IHC) staining revealed that the expression of PAHAH1B3 and the EMT markers N-cadherin and Vimentin were downregulated in si-PAHFAH1B3 xenograft tumors (Figure 7f), whereas E-cadherin expression was higher than that in the PBS and NC groups. These data confirm that PAFAH1B3 suppresses the malignant progression of HCC in vitro and in vivo by regulating EMT.

## 3. Discussion

Since the discovery of eccDNA in 1964, significant breakthroughs have been achieved in this field. Recent studies have confirmed that eccDNA is closely related to the occurrence and development of cancer [27,28,29,30]. It promotes the expression of oncogenes and harbors oncogenes with higher transcriptional activity. Furthermore, eccDNA is associated with tumor resistance during tumor drug treatment. These findings reveal the biological mechanisms underlying tumorigenesis and development, providing new insights and targets for cancer research and therapy. However, research on eccDNA in HCC is limited. Using Circle-seq, we investigated the eccDNA profiles of five pairs of HCC and para-cancerous normal tissues and analyzed their size distribution, chromosomal position, and expression levels, providing a scientific basis for further understanding of eccDNA profiles in liver cancer tissues. The gene annotation analysis showed that 45,564 eccDNAs (49.54%) could be mapped to protein-coding gene regions, with 24,513 in liver cancer tissues and 21,051 in adjacent non-cancerous tissues, demonstrating that approximately 50% of eccDNAs can be mapped to protein-coding gene regions, which is consistent with findings from previous studies [31]. The number of eccDNA loci differs substantially among chromosomes, with chromosome 19 being the most prevalent and the Y chromosome the least common [32]. In our study, the selected molecule, eccDNA PAFAH1B3, also originated from chromosome 19. The eccDNA sequencing and bioinformatics analysis revealed that eccDNA PAFAH1B3 (chr19:42803212-42803548) was significantly amplified in HCC and may be a potential biomarker in HCC patients. In previous studies, researchers found that treating immune cells with artificially synthesized circular DNAs of similar size to eccDNAs generated with random sequences reproduced the strong stimulatory capacity of purified natural eccDNAs on immune cells. Compared with linear DNA of the same concentration and length, eccDNA and synthetic circular DNA were more effective in stimulating immune responses. Notably, LAMA was recently used to create small circular DNA molecules (<1 kb) [33]. We adopted this approach and successfully obtained the artificial DNA circle PAFAH1B3. We initially transfected the artificial eccDNA PAFAH1B3, and its level was significantly increased. We found that eccDNA overexpression promoted the proliferation, migration, and invasion of liver cells while inhibiting its apoptosis.

Previous studies have shown that eccDNAs may affect gene expression by interfering with the transcription of specific exons [5,11]. EccDNAs that contain segments of MYCN facilitate the amplification of MYCN in neuroblastoma through the regulation of genome remodeling [11]. In contrast, eccDNAs with TAOK2 intronic fragments inhibit the expression of TAOK2 [34]. Reports reveal that eccDNAs may contribute to the differentially expressed genes in gastric cancer. EccDNAs carrying functional genomic segments were associated with the carcinogenesis of gastric cancer and showed the potential to facilitate cancer progression [27]. Our results reveal that after the overexpression of eccDNA PAFAH1B3 in cell lines, the protein and mRNA levels of PAFAH1B3 also increased. EccDNA PAFAH1B3 may enhance the proliferation, migration, and invasion of liver cancer cells while inhibiting apoptosis by promoting the expression of PAFAH1B3. In this study, we further investigated the role of PAFAH1B3 in HCC using in vivo and in vitro experiments. Furthermore, PAFAH1B3 has been implicated as a dysregulated metabolic gene associated with various cancer processes, including cell proliferation, apoptosis, and metastasis [35,36]. Its expression is crucial in hypopharyngeal non-squamous cell carcinoma as it modulates cell proliferation, migration, invasion, apoptosis, and cell cycle disruption [37]. Analysis of public databases validated the elevated PAFAH1B3 expression in lung adenocarcinoma and positioned PAFAH1B3 as a promising candidate for prognostic marker and potential therapeutic target in lung cancer treatment [38]. Most importantly, the researchers proved that the mRNA expression of PAFAH1B3 was also increased in HCC cell lines. PAFAH1B3 manipulates apoptosis and cell cycle regulation; silencing it inhibits the proliferation, invasion, and migration of HCC cells. Additionally, PAFAH1B3 silencing significantly downregulates the glycolysis and lipid synthesis signaling pathways [39]. To the best of our knowledge, no in vivo studies on the role of PAFAHB3 in HCC have been performed.

Tumor metastasis is a complex and multistage process that primarily encompasses the phases of invasion and dissemination [12]. During the invasion, tumor cells present within the original tumor site amplify their invasiveness by undergoing EMT [40]. PAFAH1B3 is linked to the metastasis of thyroid papillary cancer via the EMT pathway [25]. In a previous study, the knockdown of PAFAH1B3 increased and decreased the levels of the epithelial marker E-cadherin and the mesenchymal marker N-cadherin, respectively, in vitro and in vivo [26]. In our study, PAFAH1B3 silencing significantly upregulated E-cadherin expression; however, the levels of N-cadherin and vimentin decreased. After transfection with eccDNA-PAFAH1B3, the protein and mRNA levels of PAFAH1B3, N-cadherin, and vimentin were upregulated, whereas those of E-cadherin were downregulated. To investigate whether si-PAFAH1B3 could be a promising therapeutic target, we constructed xenograft tumor models of nude mice subcutaneously injecting them with SMMC-7721 cells. These data confirm that PAFAH1B3 suppresses the malignant progression of HCC in vitro and in vivo by regulating EMT.

There has been significant progress regarding the understanding of eccDNAs; however, our understanding of eccDNAs remains insufficient, and many questions still require further investigation: What are the exact mechanisms of eccDNAs formation? Can eccDNA be transcribed and translated into regulatory factors that promote oncogenic expression? Can eccDNAs molecules serve as signaling molecules that mediate intercellular communication? These points are worthy of consideration and exploration. Hence, additional research is required to clarify the specific roles of eccDNAs carrying particular gene segments in either enhancing or repressing the expression of target genes, leading to a deeper understanding of the pathogenic mechanisms of eccDNAs in cancer and thereby providing a scientific basis for disease treatment.

## 4. Materials and Methods

### 4.1. Clinical Specimens

In this study, five pairs of HCC and para-cancerous tissues were collected in 2022 and subjected to Circle-Sequencing (Circle-seq). The clinical information of the five patients of HCC was recorded (Table S5). The clinical samples above were pathologically confirmed as HCC and were obtained from Peking Union Medical College Hospital under the approval of the Hospital Ethical Committee (IRB:I-22PJ1006). Informed consent to use and publish clinical information for research purposes was obtained from all of the patients in accordance with the Declaration of Helsinki.

### 4.2. Isolation, Purification, and Sequencing of eccDNAs from Liver Cancer Tissues

#### 4.2.1. Isolation and Purification of eccDNAs

This process includes cell lysis, column purification, and removal of linear DNA. Briefly, the tissue samples were homogenized, resuspended in L1 buffer (lysis buffer) containing proteinase K, and digested overnight at 50 °C. A control plasmid (pGEX-5X-2) was spiked into the sample as an internal control after complete cell lysis. The digested samples were treated with alkali and purified via a column. Subsequently, the column-purified DNA samples were digested with FastDigest MssI at 37 °C for 16 h to remove mitochondrial circular DNA. Plasmid-Safe ATP-dependent DNase was added to the samples and digested at 37 °C for 1 week, replenishing the enzyme and ATP every 24 h to completely remove residual linear DNA. The effectiveness of linear chromosomal DNA removal was determined based on the quantitative polymerase chain reaction (qPCR) results.

Circle-seq 41: Prior to sequencing, rolling-circle amplification (RCA) and library construction were performed. The purified samples were used as templates for RCA using the RCA DNA Amplification Kit (New England Biolabs, Ipswich, MA, USA). This was followed by purification using the MinElute Reaction Cleanup Kit (QIAGEN GmbH, Hilden, North Rhine-Westphalia, Germany). The amplified products were sonicated to fragment sizes of approximately 150 bp. Furthermore, library construction was performed using the GenSeq^®^ Rapid DNA Lib Prep Kit (ABC Biolabs, Woburn, MA, USA). As previously reported, the libraries were subjected to high-throughput sequencing on a NovaSeq 6000 platform (Illumina Inc., San Diego, CA, USA) using 150 bp paired-end mode.

#### 4.2.2. Bioinformatics Analysis and Screening of Differentially Expressed eccDNAs in Liver Cancer Tissues

Bioinformatics analysis: Sequencing was performed using the Illumina NovaSeq 6000 platform to obtain raw data. Initial quality control of raw data was assessed using Q30 values. Adaptor trimming and removal of low-quality reads were performed using cutadapt (v.1.9.1) to obtain high-quality clean reads. The clean reads were then aligned to the reference genome using bwa (v0.7.12). Subsequently, circle-map (v1.1.4) was employed to identify eccDNAs across all samples. Samtools (v1.9) was used to count soft-clipped reads overlapping breakpoints as raw counts. Differential eccDNA expression analysis was performed using DESeq2 (v1.34.0) for normalization, calculation of fold-changes and *p*-values between sample groups, and screening of differentially expressed eccDNAs. Gene annotation of identified eccDNAs and differentially expressed eccDNAs was conducted using bedtools (v2.27.1). Differentially expressed genes and their intersections in Sk-hep1 and Huh7 cells transfected with eccDNA PAFAH1B3 are shown in the Venn diagram (https://bioinfogp.cnb.csic.es/tools/venny/, accessed on 1 April 2023). The “clusterProfiler” R package(v4.2.1) was utilized for GO and KEGG analyses, with q- and *p*-value thresholds set at *p* < 0.05.

#### 4.2.3. Screening of Differentially Expressed eccDNAs

We downloaded the transcriptome data for liver cancer (374 cases) and control tissues (50 cases) from the TCGA database. Differentially expressed mRNAs between liver cancer and control tissues were analyzed using the DESeq2 package in R (v4.0.3). The criteria for significant differential expression were set as |log2(fold change)| > 1 and adjusted *p*-value < 0.05 and gene annotation was performed for these mRNAs. The intersection between the differentially expressed genes identified in the sequencing analysis and those from the TCGA database that were significant in at least three tissues were obtained. Furthermore, the GEPIA2 database was used to examine the potential role of the intersected genes in liver cancer prognosis assessment. The technical roadmap for screening eccDNAs that regulate the occurrence and development of liver cancer is shown in Figure S1. Ultimately, eccDNA PAFAH1B3 was selected as one of the research targets for further investigation.

#### 4.2.4. Quantification of eccDNA PAFAH1B3 Expression in Liver Cancer Tissues and Circularity Validation

We used the outward-facing primer design strategy to target the specific junction site of eccDNA PAFAH1B3. Primers 1 and 2 are designed to be tail-to-tail for linear DNA, which prevents efficient amplification. However, if the linear DNA forms a closed circular DNA by joining the ends, primers 1 and 2 can amplify the product specific to the junction site (Figure S3). The primer sequences include eccDNA PAFAH1B3 Forward: ATGGTGCAATCTTGGCTCAC, Reverse: AAAATTAGCTGGGCGTGGTG; pGEX-5X-2 Forward: ATGGTGCAATCTTGGCTCAC, Reverse: AAAATTAGCTGGGCGTGGTG.

#### 4.2.5. Circularity Validation of eccDNA PAFAH1B3

Agarose gel electrophoresis: We performed inward and outward polymerase chain reaction (PCR) targeting of eccDNA PAFAH1B3 using genomic DNA, enriched eccDNAs, RCA-amplified eccDNAs, and a no-template negative control. The amplified products were subsequently analyzed using agarose gel electrophoresis to confirm the presence of eccDNA PAFAH1B3.

Sanger sequencing: The amplified products covering the junction sites of eccDNA PAFAH1B3 were subjected to Sanger sequencing to accurately validate the presence of the junction sites. The circular junction sites of eccDNA PAFAH1B3 were confirmed through low-throughput Sanger sequencing, providing further evidence for the circularity of eccDNA PAFAH1B3 and validating the accuracy of high-throughput sequencing analysis in identifying eccDNA PAFAH1B3.

#### 4.2.6. Artificial Circular DNA Synthesis

We created artificial DNA circles of PAFAH1B3 by the procedure of ligase-assisted minicircle accumulation (LAMA)34. We identified the endogenous eccDNA sequence fragments of interest; then, PCR amplification primers were designed on the genome based on the start and end sites of eccDNA, and the type II restriction enzyme recognition site sequence was added. The oligonucleotide sequences sense strand (SS) and antisense strand (AS) of linker DNA were designed and synthesized; SS and AS were complementarity joined with double stranded DNA with sticky ends through high-temperature denaturation and low-temperature annealing; the amplified product was mixed with linker DNA from the same endogenous eccDNA binding site; enzyme digestion ligation cycle reaction was performed on the mixture to obtain sufficient eccDNA. Subsequently, the linear fragments were removed from the reaction system. After purification, seamless connected eccDNA in line with the endogenous sequence information can be obtained.

### 4.3. EdU Assays

Cell proliferation was assessed using the EdU (5-ethynyl-2′-deoxyuridine) assay kit (Beyotime Biotechnology, Haimen, Jiangsu, China) following the manufacturer’s instructions. SK-Hep1 and Huh7 cells were seeded in 24-well plates using a density of 1 × 10^5 cells/well in a complete medium and transfected with NC, eccDNA PAFAH1B3. After incubating for 24 h, the cells were fixed with 4% paraformaldehyde for 15 min at room temperature and further permeabilized with 0.3% Triton X-100 in phosphate-buffered saline (PBS) for 10 min. The cells were incubated with the EdU labeling solution following instructions provided with the kit, washed with PBS to remove excess EdU, and counterstained with DAPI (4′,6-diamidino-2-phenylindole) to stain the DNA. The stained cells were observed under a fluorescence microscope (Olympus or similar, Kita-ku, Tokyo 108-0072, Japan) using the FITC channel for EdU.

### 4.4. Transwell Cell Migration and Invasion Assays

The migration and invasion assays were conducted utilizing Transwell plates, with or without Matrigel coating in the upper chambers. SK-Hep1 and Huh7 cells were transfected with negative control (NC) and eccDNA PAFAH1B3, and then incubated in Transwell plates for 24 h. The cells were digested with 0.25% trypsin, resuspended in a serum-free medium, and subsequently counted. A volume of 200 μL of the transfected cells was seeded into the upper chamber, while 600 μL of Dulbecco’s Modified Eagle Medium (DMEM) supplemented with 10% fetal bovine serum (FBS) was added to the lower chamber. Following a 24 h incubation period, the upper chamber was washed twice with phosphate-buffered saline (PBS). Cells on the upper surface were removed using a cotton swab, and the remaining cells were fixed with anhydrous methanol and stained with 0.1% crystal violet for 30 min. Cells that migrated through the 8 μm pore membrane or invaded through the Matrigel-coated membrane were subsequently stained.

### 4.5. Apoptosis Assays

In summary, cells were subjected to digestion using trypsin devoid of ethylenediaminetetraacetic acid (EDTA) and subsequently rinsed with ice-cold PBS. Apoptotic activity was assessed through dual staining with fluorescein allophycocyanin-conjugated Annexin V and propidium iodide (PI). Subsequently, the cell populations were quantified utilizing a flow cytometer (BD Accuri C6 Plus, Franklin Lakes, NJ, USA). Data analysis and visualization were conducted using FlowJo software(v 10.8.1).

### 4.6. RNA Extraction and Reverse Transcription–Quantitative Polymerase Chain Reaction

We extracted total RNA using the Trizol reagent (Invitrogen, Carlsbad, CA, USA) following the manufacturer’s instructions. A NanoDrop ND-2000 instrument (Thermo Fisher Scientific, Waltham, MA, USA) was used to evaluate the quality of the RNA samples. Total RNA was isolated, and 2 μg of it was used in reverse transcription with PrimeScript RT Reagent Kit (Takara, Dalian, China) based on the manufacturer’s protocol. Reverse transcription–quantitative polymerase chain reaction (RT-qPCR) was conducted on the ABI Prism 7500 system (Applied Biosystems, Carlsbad, CA, USA) with SYBR Green Premix Ex Taq (Takara, Dalian, China). Glyceraldehyde-3-phosphate dehydrogenase was used as an internal control. The relative expression of genes was calculated using the 2-ΔΔCT method and the primers used in the experiments were purchased from S Sangon Biotech (Shanghai, China) Co., Ltd. and are presented in Table S6.

### 4.7. Western Blot Analysis

Cells and tissues were treated with an appropriate volume of radioimmunoprecipitation assay (RIPA) buffer containing phenylmethylsulfonyl fluoride. Subsequently, total protein was extracted via centrifugation, and its concentration was quantified using the bicinchoninic acid (BCA) assay. Equal amounts of total protein (20 μg) were resolved by 8% sodium dodecyl sulfate–polyacrylamide gel electrophoresis (SDS-PAGE) and transferred onto polyvinylidene fluoride (PVDF) membranes (Millipore, Billerica, MA, USA). To block nonspecific binding, the membranes were incubated with 5% skim milk for 1 h at room temperature. The membranes were then incubated overnight at 4 °C with the following primary antibodies: PAFAH1B3 (Proteintech, Rosemont, Illinois, USA, 20564-1-AP), E-cadherin (Abcam, Cambridge, UK, ab40772), N-cadherin (Abcam, Cambridge, UK, ab76011), and Vimentin (Abcam, Cambridge, UK, ab92547), with the latter serving as the internal control. After washing five times with 1× TBST for 5 min each, the membranes were incubated with horseradish peroxidase (HRP)-conjugated secondary antibodies (goat anti-rabbit IgG or goat anti-mouse IgG) at room temperature for 1 h. The membranes were washed again five times with 1× TBST for 5 min each. Protein bands were visualized using a chemiluminescence imaging system (SageCreation Science, Beijing, China) with SuperSignal™ West Femto Maximum Sensitivity Substrate (Thermo Scientific, Waltham, MA, USA).

### 4.8. Xenograft Tumorigenesis

Fifteen specific pathogen-free (SPF)-grade male BALB/C nude mice, aged 6 to 8 weeks, were procured from Beijing Vital River Laboratory Animal Technology Co., Ltd. (Beijing, China). The average weight of the animals was 15.5 g, with a range of 14.5 to 16.0 g. All nude mice were maintained under SPF conditions (TECNIPLAST S.p.A., Milan, Italy) and were randomly assigned to three experimental groups. A total of 5 × 10^6 SMMC-7721 cells were suspended in 100 μL of PBS and subcutaneously injected into the right flank of each mouse. Upon reaching a tumor volume of 100–200 mm^3^, intratumoral injections of PBS, cholesterol-conjugated NC, and si-PAFAH1B3 were administered five times at three-day intervals. Tumor volume was measured every three days using the formula V (volume) = (length × width^2^)/2. Tumor weight was recorded at the conclusion of the experiment. After 24 days, the mice were euthanized, and the tumors were excised and prepared for histological analysis.

### 4.9. Immunohistochemistry Staining

Paraffin-embedded sections of xenograft tumor tissues were sliced into 4 μm thick sections. These sections underwent a series of preparatory steps, including dewaxing, rehydration, antigen retrieval, and blocking. They were then incubated with antibodies targeting E-cadherin, N-cadherin, and Vimentin for 70 min at room temperature within a humidified chamber. This was followed by five washes with PBS. Subsequently, the sections were incubated with an HRP-conjugated secondary antibody for 20 min at room temperature and again washed five times with PBS. The sections were then stained with diaminobenzidine and counterstained with hematoxylin. Finally, the sections were dehydrated and mounted with coverslips for analysis.

### 4.10. Statistical Analysis

The Kolmogorov–Smirnov test was used to estimate the distribution of the data. Normally distributed continuous variables are presented as mean ± standard deviation values. Statistical significance was determined using a one-way analysis of variance test or Student’s *t*-test. Data were processed with GraphPad (San Diego, CA, USA), and all *p* values presented are two-tailed, and statistical significance was set at *p* < 0.05. Analyses were performed using the Statistical Package for Social Sciences 20.0 software (Armonk, NY, USA).

## 5. Conclusions

Our study provides comprehensive insights into the eccDNAs landscape of HCC and highlights the importance of these molecules in cancer biology. Identifying key eccDNAs and their association with critical oncogenes, such as PAFAH1B3, lays the groundwork for future research aimed at targeting EMT in HCC and other malignancies. As we continue to unravel the complexities of eccDNA functions, we anticipate that our findings will significantly contribute to the understanding of cancer pathogenesis and therapeutic development.

## Data Availability

The datasets used and analyzed during the current study are available from the corresponding author on reasonable request.

## Supplementary Materials

The following supporting information can be downloaded at https://www.mdpi.com/article/10.3390/ijms26188801/s1.

## Abbreviations

The following abbreviations are used in this manuscript:

EccDNA	Extrachromosomal circular DNAs
HCC	Hepatocellular carcinoma
EMT	Epithelial–mesenchymal transition
PAFAH1B3	Platelet-activating factor acetylhydrolase 1B3
NSCLC	Non-small cell lung cancer
