# Supplementary material for: PAFAH1B3 Exists in Linear Chromosomal and Extrachromosomal Circular DNA and Promotes HCC Progression via EMT
Source: Int J Mol Sci. 2025 Sep 10;26(18):8801. doi: 10.3390/ijms26188801 (PMC12469353; doi:10.3390/ijms26188801)
Supplement: Supplementary file 1 [file ijms-26-08801-s001.zip › Supplementary Figure legends.pdf]

Fig.S1 Analysis of PAFAH1B3 expression via RT-qPCR in HepG2 (a) and Huh7 (b) cells.

Fig.S2 Technical roadmap for screening eccDNAs that regulate the occurrence and development of HCC.

Fig.S3 Schematic diagram of eccDNA PAFAH1B3 primer design.
