# Supplementary material for: PAFAH1B3 Exists in Linear Chromosomal and Extrachromosomal Circular DNA and Promotes HCC Progression via EMT
Source: Int J Mol Sci. 2025 Sep 10;26(18):8801. doi: 10.3390/ijms26188801 (PMC12469353; doi:10.3390/ijms26188801)
Supplement: Supplementary file 1 [file ijms-26-08801-s001.zip › Supplementary Table 1.pdf]

Table S1 The upregulated differentially expressed eccDNAs detected in at least three pairs of samples,of which 21 were mapped to protein-coding genes

ecc_chr	ecc_start	ecc_end	ecc_location	gene_name	gene_biotype
chr15	90386428	90386674	chr15:90386428-90386674	AP3S2	protein_coding
chr1	71060980	71061415	chr1:71060980-71061415		
chr18	5004075	5004705	chr18:5004075-5004705		
chr1	205720819	205721179	chr1:205720819-205721179		
chr4	96893567	96894304	chr4:96893567-96894304		
chr9	111313703	111314358	chr9:111313703-111314358		
chr15	41538860	41539205	chr15:41538860-41539205	CHP1	protein_coding
chr22	39987002	39989075	chr22:39987002-39989075	CACNA1I	protein_coding
chr8	68766505	68767704	chr8:68766505-68767704		
chr14	68915258	68918646	chr14:68915258-68918646	RAD51B	protein_coding
chr1	201582470	201582879	chr1:201582470-201582879		
chr2	15358032	15359747	chr2:15358032-15359747	NBAS	protein_coding
chr11	122236865	122241388	chr11:122236865-122241388		
chr21	9930059	9930514	chr21:9930059-9930514		
chr6	31313774	31315181	chr6:31313774-31315181		
chr22	18172429	18173321	chr22:18172429-18173321	BCL2L13	protein_coding
chr6	124225251	124233359	chr6:124225251-124233359	NKAIN2	protein_coding
chr7	28074550	28080354	chr7:28074550-28080354	JAZF1	protein_coding
chr4	123358838	123359062	chr4:123358838-123359062		
chr19	52921609	52925209	chr19:52921609-52925209	ZNF528	protein_coding
chr15	41883771	41885749	chr15:41883771-41885749		
chr11	134061795	134063260	chr11:134061795-134063260	NCAPD3	protein_coding
chr19	42803212	42803549	chr19:42803212-42803549	PAFAH1B3	protein_coding
chr2	238796880	238799399	chr2:238796880-238799399	RAMP1	protein_coding
chr1	6601509	6601809	chr1:6601509-6601809	NOL9	protein_coding
chr6	42815302	42815602	chr6:42815302-42815602	GLTSCR1L	protein_coding
chr12	114251653	114255494	chr12:114251653-114255494	RBM19	protein_coding
chr7	75423656	75424257	chr7:75423656-75424257		
chr6	64287380	64287780	chr6:64287380-64287780	PTP4A1	protein_coding
chr8	111109921	111116487	chr8:111109921-111116487		

chr16	88692306	88693579	chr16:88692306-88693579	ZC3H18	protein_coding
chr9	133448905	133449955	chr9:133448905-133449955		
chr6	42878070	42878631	chr6:42878070-42878631		
chr8	12930508	12931092	chr8:12930508-12931092		
chr13	79951286	79952651	chr13:79951286-79952651	RBM26	protein_coding
chr16	30474217	30475875	chr16:30474217-30475875		
chr3	47275696	47281475	chr3:47275696-47281475	KIF9	protein_coding
chrX	56041795	56044435	chrX:56041795-56044435		
chr1	36186091	36186396	chr1:36186091-36186396	CLSPN	protein_coding
chr6	44770471	44772428	chr6:44770471-44772428		
chr19	54884496	54887384	chr19:54884496-54887384		
chr20	3223949	3224367	chr20:3223949-3224367		
chr20	34846668	34848811	chr20:34846668-34848811	AAR2	protein_coding
chr15	70131959	70133687	chr15:70131959-70133687		
