# Supplementary material for: PAFAH1B3 Exists in Linear Chromosomal and Extrachromosomal Circular DNA and Promotes HCC Progression via EMT
Source: Int J Mol Sci. 2025 Sep 10;26(18):8801. doi: 10.3390/ijms26188801 (PMC12469353; doi:10.3390/ijms26188801)
Supplement: Supplementary file 1 [file ijms-26-08801-s001.zip › Supplementary Table 2.pdf]

Table S2 The downregulated differentially expressed eccDNAs were detected in at least three pairs of samples,of which 211 were mapped to protein-coding genes

ecc_chr	ecc_start	ecc_end	ecc_location	gene_name	gene_biotype
chr11	134597470	134597768	chr11:134597470-134597768		
chr18	21342584	21342885	chr18:21342584-21342885	LAMA3	protein_coding
chr1	92081510	92081823	chr1:92081510-92081823		
chr10	86671940	86672277	chr10:86671940-86672277		
chr12	116805379	116805780	chr12:116805379-116805780		
chr17	37143213	37143656	chr17:37143213-37143656		
chr20	30117277	30117892	chr20:30117277-30117892	HM13	protein_coding
chr7	34015685	34016513	chr7:34015685-34016513	BMPER	protein_coding
chr7	136191091	136192228	chr7:136191091-136192228		
chr22	41793760	41795208	chr22:41793760-41795208	TEF	protein_coding
chr11	101772951	101774922	chr11:101772951-101774922	ANGPTL5	protein_coding
chr2	148643802	148645925	chr2:148643802-148645925	ACVR2A	protein_coding
chr15	96413830	96416363	chr15:96413830-96416363		
chr15	24718238	24721043	chr15:24718238-24721043		
chr15	48513016	48516353	chr15:48513016-48516353	SLC12A1	protein_coding
chr4	118072733	118076139	chr4:118072733-118076139		
chr18	32896465	32901138	chr18:32896465-32901138		
chr6	152653095	152657879	chr6:152653095-152657879	SYNE1	protein_coding
chrX	30762886	30768241	chrX:30762886-30768241		
chr2	226217386	226223451	chr2:226217386-226223451		
chr3	109026030	109026256	chr3:109026030-109026256	DPPA2	protein_coding
chr17	74600428	74600881	chr17:74600428-74600881		
chr22	45408994	45409784	chr22:45408994-45409784		
chr12	6180414	6182865	chr12:6180414-6182865	VWF	protein_coding
chr11	100883618	100887878	chr11:100883618-1008878		

			78		
chr8	41253613	41258229	chr8:41253613-41258229		
chr1	153806297	153806467	chr1:153806297-153806467	GATAD2B	protein_coding
chr20	62341618	62341920	chr20:62341618-62341920	ZGPAT	protein_coding
chr16	15092918	15093227	chr16:15092918-15093227	PDXDC1	protein_coding
chr17	26745973	26746289	chr17:26745973-26746289		
chr15	76195207	76195539	chr15:76195207-76195539		
chr19	17457257	17457624	chr19:17457257-17457624		
chr7	65194005	65194379	chr7:65194005-65194379		
chr16	15062822	15063212	chr16:15062822-15063212		
chr19	45918800	45919332	chr19:45918800-45919332	ERCC1	protein_coding
chr7	97894391	97894937	chr7:97894391-97894937	BRI3	protein_coding
chr7	148614658	148615211	chr7:148614658-148615211		
chr10	114828632	114829202	chr10:114828632-114829202	TCF7L2	protein_coding
chr20	18550486	18551072	chr20:18550486-18551072		
chr6	111603451	111604081	chr6:111603451-111604081		
chr14	30679650	30680475	chr14:30679650-30680475		
chr4	183936460	183937585	chr4:183936460-183937585		
chr10	102602863	102604083	chr10:102602863-102604083		
chr3	73743661	73745006	chr3:73743661-73745006		
chr12	53304316	53305885	chr12:53304316-53305885	KRT8	protein_coding
chr7	65461525	65463175	chr7:65461525-65463175		
chr7	102557327	102559025	chr7:102557327-102559025	LRRC17	protein_coding
chr3	40763798	40765602	chr3:40763798-40765602		
chr11	102762002	102763925	chr11:102762002-102763925		
chr16	70901426	70903560	chr16:70901426-70903560	HYDIN	protein_coding
chr15	45626677	45629410	chr15:45626677-45629410		
chr20	57606682	57609503	chr20:57606682-57609503	SLMO2	protein_coding

chr7	115483485	115486314	chr7:115483485-115486314		
chr1	195530058	195533315	chr1:195530058-195533315		
chr7	19395467	19398768	chr7:19395467-19398768		
chr22	41983649	41987613	chr22:41983649-41987613	PMM1	protein_coding
chr1	248209395	248213491	chr1:248209395-248213491	OR2L13	protein_coding
chr11	61025167	61029537	chr11:61025167-61029537	VWCE	protein_coding
chr2	124120326	124124772	chr2:124120326-124124772		
chr4	95486045	95490545	chr4:95486045-95490545	PDLIM5	protein_coding
chr2	77275179	77280033	chr2:77275179-77280033	LRRTM4	protein_coding
chr16	83589995	83595354	chr16:83589995-83595354	CDH13	protein_coding
chr10	83822118	83827615	chr10:83822118-83827615	NRG3	protein_coding
chr13	78425391	78431065	chr13:78425391-78431065		
chr12	72736653	72742466	chr12:72736653-72742466	TRHDE	protein_coding
chr10	17325861	17332278	chr10:17325861-17332278		
chr3	174250552	174257268	chr3:174250552-174257268	NAALADL2	protein_coding
chr1	248206451	248213324	chr1:248206451-248213324	OR2L13	protein_coding
chr1	35334467	35341452	chr1:35334467-35341452	DLGAP3	protein_coding
chr7	43981507	43990666	chr7:43981507-43990666	UBE2D4	protein_coding
chrX	11370854	11371020	chrX:11370854-11371020	ARHGAP6	protein_coding
chr19	4265949	4266145	chr19:4265949-4266145	CCDC94	protein_coding
chr20	31917479	31917696	chr20:31917479-31917696		
chr5	101875517	101875736	chr5:101875517-101875736		
chr14	87306462	87306694	chr14:87306462-87306694		
chr2	234249359	234249600	chr2:234249359-234249600	SAG	protein_coding

chr3	56678104	56678351	chr3:56678104-56678351	FAM208A	protein_coding
chr1	10801118	10801369	chr1:10801118-10801369	CASZ1	protein_coding
chr15	82219917	82220181	chr15:82219917-82220181		
chr11	18843864	18844136	chr11:18843864-18844136		
chr22	30950203	30950481	chr22:30950203-30950481		
chr11	14013502	14013802	chr11:14013502-14013802		
chr16	9616949	9617255	chr16:9616949-9617255		
chr16	77864171	77864478	chr16:77864171-77864478	VAT1L	protein_coding
chr11	66271057	66271366	chr11:66271057-66271366	DPP3	protein_coding
chr18	55452803	55453117	chr18:55452803-55453117	ATP8B1	protein_coding
chr15	90412916	90413252	chr15:90412916-90413252	AP3S2	protein_coding
chr3	152671580	152671920	chr3:152671580-152671920		
chr11	23540647	23540993	chr11:23540647-23540993		
chr17	37854374	37854722	chr17:37854374-37854722	ERBB2	protein_coding
chr7	30311698	30312046	chr7:30311698-30312046		
chr6	149534561	149534930	chr6:149534561-149534930		
chr5	132789234	132789608	chr5:132789234-132789608	FSTL4	protein_coding
chr4	8330008	8330389	chr4:8330008-8330389		
chr6	30606071	30606452	chr6:30606071-30606452	ATAT1	protein_coding
chr13	66875963	66876353	chr13:66875963-66876353		
chr2	160291770	160292166	chr2:160291770-160292166	BAZ2B	protein_coding
chr7	71459807	71460204	chr7:71459807-71460204	CALN1	protein_coding
chr17	31191101	31191506	chr17:31191101-31191506	MYO1D	protein_coding
chr8	92891011	92891417	chr8:92891011-92891417		
chr1	235570415	235570822	chr1:235570415-235570822	TBCE	protein_coding
chr9	16907550	16907962	chr9:16907550-16907962		
chr2	46149654	46150069	chr2:46149654-46150069	PRKCE	protein_coding

chr8	16753476	16753923	chr8:16753476-16753923		
chr10	30416473	30416937	chr10:30416473-30416937		
chr6	42049009	42049482	chr6:42049009-42049482	TAF8	protein_coding
chr19	697190	697666	chr19:697190-697666		
chr1	41137914	41138390	chr1:41137914-41138390		
chr1	37739872	37740355	chr1:37739872-37740355		
chr17	1285599	1286084	chr17:1285599-1286084	YWHAE	protein_coding
chr12	123955237	123955729	chr12:123955237-123955729	SNRNP35	protein_coding
chr17	76205486	76205989	chr17:76205486-76205989		
chr9	20884539	20885046	chr9:20884539-20885046	FOCAD	protein_coding
chr8	9975717	9976234	chr8:9975717-9976234	MSRA	protein_coding
chr1	33912337	33912863	chr1:33912337-33912863		
chr2	149184826	149185353	chr2:149184826-149185353	MBD5	protein_coding
chr16	27074208	27074746	chr16:27074208-27074746		
chr8	21039747	21040288	chr8:21039747-21040288		
chr19	14220630	14221182	chr19:14220630-14221182	PRKACA	protein_coding
chr16	59889729	59890288	chr16:59889729-59890288		
chrX	111262376	111262942	chrX:111262376-111262942	TRPC5	protein_coding
chr8	59807167	59807742	chr8:59807167-59807742	TOX	protein_coding
chr5	139816032	139816608	chr5:139816032-139816608	ANKHD1	protein_coding
chr19	23400674	23401269	chr19:23400674-23401269		
chr11	18452651	18453255	chr11:18452651-18453255	LDHC	protein_coding
chr2	202983642	202984252	chr2:202983642-202984252	AC079354.1	protein_coding
chr1	235602964	235603582	chr1:235602964-235603582	TBCE	protein_coding
chr12	88792668	88793289	chr12:88792668-88793289		
chr15	44571258	44571884	chr15:44571258-44571884		
chr20	49041626	49042255	chr20:49041626-49042255		
chr12	7901797	7902450	chr12:7901797-7902450	CLEC4C	protein_coding
chr11	63709442	63710104	chr11:63709442-63710104	NAA40	protein_codin

					g
chr5	174138930	174139605	chr5:174138930-174139605		
chr2	85319170	85319854	chr2:85319170-85319854		
chr3	104244079	104244764	chr3:104244079-104244764		
chr10	15237433	15238120	chr10:15237433-15238120		
chr16	78137098	78137788	chr16:78137098-78137788	WWOX	protein_coding
chrX	153331694	153332416	chrX:153331694-153332416	MECP2	protein_coding
chr19	19478396	19479125	chr19:19478396-19479125		
chr3	24333997	24334731	chr3:24333997-24334731	THRB	protein_coding
chr6	37733651	37734386	chr6:37733651-37734386		
chr8	21735996	21736741	chr8:21735996-21736741		
chr9	124766153	124766899	chr9:124766153-124766899	TTLL11	protein_coding
chr9	89738243	89738993	chr9:89738243-89738993		
chr19	47741821	47742581	chr19:47741821-47742581		
chr18	20634986	20635788	chr18:20634986-20635788		
chr8	50222149	50222955	chr8:50222149-50222955		
chr11	126031577	126032394	chr11:126031577-126032394		
chr4	46912401	46913266	chr4:46912401-46913266		
chr4	118906313	118907195	chr4:118906313-118907195		
chr2	106554345	106555236	chr2:106554345-106555236		
chr8	68889158	68890052	chr8:68889158-68890052	PREX2	protein_coding
chr21	27683107	27684007	chr21:27683107-27684007		
chr19	34132705	34133640	chr19:34132705-34133640	CHST8	protein_coding
chr11	106507354	106508295	chr11:106507354-106508295		
chrX	10427317	10428274	chrX:10427317-10428274	MID1	protein_coding
chr20	33663538	33664540	chr20:33663538-33664540	TRPC4AP	protein_coding
chr20	14117261	14118276	chr20:14117261-14118276	MACROD2	protein_coding
chr17	70804025	70805092	chr17:70804025-70805092	SLC39A11	protein_codin

					g
chr8	145232508	145233615	chr8:145232508-145233615	MROH1	protein_coding
chr3	81676977	81678142	chr3:81676977-81678142	GBE1	protein_coding
chr8	6395998	6397166	chr8:6395998-6397166	ANGPT2	protein_coding
chr1	154952737	154953934	chr1:154952737-154953934		
chr4	118035626	118036831	chr4:118035626-118036831		
chr7	26214674	26215904	chr7:26214674-26215904	NFE2L3	protein_coding
chr5	121709901	121711221	chr5:121709901-121711221	SNCAIP	protein_coding
chr4	129000409	129001796	chr4:129000409-129001796	LARP1B	protein_coding
chr7	95492408	95493799	chr7:95492408-95493799	DYNC1I1	protein_coding
chr10	96346034	96347471	chr10:96346034-96347471	HELLS	protein_coding
chr3	53241638	53243137	chr3:53241638-53243137		
chr11	37632052	37633560	chr11:37632052-37633560		
chr2	116233662	116235201	chr2:116233662-116235201	DPP10	protein_coding
chr3	192114887	192116472	chr3:192114887-192116472	FGF12	protein_coding
chr5	149920148	149921782	chr5:149920148-149921782	NDST1	protein_coding
chr6	71092464	71094111	chr6:71092464-71094111		
chr13	66085079	66086727	chr13:66085079-66086727		
chr4	7130537	7132187	chr4:7130537-7132187		
chr3	56152169	56153822	chr3:56152169-56153822	ERC2	protein_coding
chr1	48547885	48549545	chr1:48547885-48549545		
chr20	46907142	46908818	chr20:46907142-46908818		
chr5	92408687	92410464	chr5:92408687-92410464		
chr3	173646960	173648765	chr3:173646960-173648765	NLGN1	protein_coding
chr14	93304576	93306410	chr14:93304576-93306410	GOLGA5	protein_coding
chr12	12920456	12922294	chr12:12920456-12922294	APOLD1	protein_coding

chr7	140359121	140360966	chr7:140359121-140360966	DENND2A	protein_coding
chr21	34736958	34738847	chr21:34736958-34738847		
chr13	42061666	42063556	chr13:42061666-42063556		
chr2	171318054	171319945	chr2:171318054-171319945	MYO3B	protein_coding
chr3	4347681	4349584	chr3:4347681-4349584	SETMAR	protein_coding
chr10	111180971	111182894	chr10:111180971-111182894		
chr16	7418150	7420111	chr16:7418150-7420111	RBFOX1	protein_coding
chr12	60678377	60680375	chr12:60678377-60680375		
chr3	9467302	9469352	chr3:9467302-9469352	SETD5	protein_coding
chr7	131936729	131938785	chr7:131936729-131938785	PLXNA4	protein_coding
chr3	48818558	48820659	chr3:48818558-48820659	PRKAR2A	protein_coding
chr12	90477552	90479661	chr12:90477552-90479661		
chr2	240663236	240665390	chr2:240663236-240665390		
chr10	44098452	44100646	chr10:44098452-44100646		
chr14	90492238	90494461	chr14:90492238-90494461	TDP1	protein_coding
chr12	103994029	103996309	chr12:103994029-103996309	STAB2	protein_coding
chr6	105131133	105133445	chr6:105131133-105133445		
chr10	103889984	103892317	chr10:103889984-103892317		
chr1	192388432	192390867	chr1:192388432-192390867		
chr18	36257623	36260066	chr18:36257623-36260066		
chr10	69484947	69487431	chr10:69484947-69487431		
chr8	118010325	118012892	chr8:118010325-118012892	SLC30A8	protein_coding
chr9	139394183	139396752	chr9:139394183-139396752	NOTCH1	protein_coding
chr8	86878033	86880612	chr8:86878033-86880612		
chr1	82111434	82114090	chr1:82111434-82114090	LPHN2	protein_coding
chr5	100144190	100146933	chr5:100144190-10014693	ST8SIA4	protein_codin

			3		g
chr12	63711677	63714524	chr12:63711677-63714524		
chr6	75066343	75069392	chr6:75066343-75069392		
chr14	40474713	40477845	chr14:40474713-40477845		
chr11	14588685	14591873	chr11:14588685-14591873	PSMA1	protein_coding
chr2	96166620	96169830	chr2:96166620-96169830		
chr11	93917729	93920944	chr11:93917729-93920944		
chr4	10048301	10051537	chr4:10048301-10051537	SLC2A9	protein_coding
chr3	98804103	98807350	chr3:98804103-98807350		
chr16	52831400	52834698	chr16:52831400-52834698		
chr6	35171949	35175314	chr6:35171949-35175314		
chr4	136055522	136058930	chr4:136055522-136058930		
chr5	86705469	86709033	chr5:86705469-86709033	CCNH	protein_coding
chr16	13137003	13140745	chr16:13137003-13140745	SHISA9	protein_coding
chr5	179792587	179796343	chr5:179792587-179796343		
chr18	32661841	32665612	chr18:32661841-32665612	MAPRE2	protein_coding
chr6	78640478	78644311	chr6:78640478-78644311		
chr18	50656901	50660843	chr18:50656901-50660843	DCC	protein_coding
chr8	68643202	68647190	chr8:68643202-68647190	CPA6	protein_coding
chr20	38167700	38171872	chr20:38167700-38171872		
chr13	49014833	49019084	chr13:49014833-49019084	LPAR6	protein_coding
chr11	119521833	119526191	chr11:119521833-119526191	PVRL1	protein_coding
chr7	26628754	26633162	chr7:26628754-26633162		
chr1	39937208	39941705	chr1:39937208-39941705	MACF1	protein_coding
chr11	4872411	4876986	chr11:4872411-4876986	MMP26	protein_coding
chr6	26510645	26515243	chr6:26510645-26515243	BTN1A1	protein_coding
chr12	42996436	43001112	chr12:42996436-43001112		
chr22	42580304	42585086	chr22:42580304-42585086	TCF20	protein_coding

chr12	71137600	71142418	chr12:71137600-71142418	PTPRR	protein_coding
chr2	2774833	2779806	chr2:2774833-2779806		
chr5	6788071	6793498	chr5:6788071-6793498		
chr4	24400771	24406257	chr4:24400771-24406257		
chr6	49536098	49541752	chr6:49536098-49541752		
chr3	140306178	140312048	chr3:140306178-140312048		
chr13	36557283	36563827	chr13:36557283-36563827	DCLK1	protein_coding
chr3	62499264	62505869	chr3:62499264-62505869	CADPS	protein_coding
chr10	3662143	3668981	chr10:3662143-3668981		
chr1	214463937	214471694	chr1:214463937-214471694	SMYD2	protein_coding
chrX	42305998	42313806	chrX:42305998-42313806		
chr8	103046804	103054956	chr8:103046804-103054956	NCALD	protein_coding
chr14	26592114	26602176	chr14:26592114-26602176		
chr6	29957410	29968567	chr6:29957410-29968567		
chr5	90384813	90385107	chr5:90384813-90385107	GPR98	protein_coding
chr20	60725946	60726265	chr20:60725946-60726265	SS18L1	protein_coding
chr1	184958705	184959058	chr1:184958705-184959058		
chr20	55227506	55227880	chr20:55227506-55227880		
chr12	6695032	6695455	chr12:6695032-6695455	CHD4	protein_coding
chr11	61877720	61878316	chr11:61877720-61878316		
chr9	81919339	81919960	chr9:81919339-81919960		
chr8	37954261	37955273	chr8:37954261-37955273		
chr1	241750176	241751237	chr1:241750176-241751237	KMO	protein_coding
chr1	244912617	244913682	chr1:244912617-244913682		
chr9	12657246	12658385	chr9:12657246-12658385		
chr20	31929033	31930473	chr20:31929033-31930473		
chr20	50677579	50679082	chr20:50677579-50679082	ZFP64	protein_coding
chr12	24814091	24815779	chr12:24814091-24815779		
chr10	97619097	97620933	chr10:97619097-97620933	RP11-248J23.7	protein_coding

chr4	177181810	177183727	chr4:177181810-177183727	ASB5	protein_coding
chr15	97428760	97431002	chr15:97428760-97431002		
chr6	42870984	42873233	chr6:42870984-42873233		
chr22	34956148	34958595	chr22:34956148-34958595		
chr8	146043938	146046479	chr8:146043938-146046479		
chr12	130124787	130127574	chr12:130124787-130127574	TMEM132D	protein_coding
chr12	79884112	79887349	chr12:79884112-79887349		
chr21	40135743	40139025	chr21:40135743-40139025		
chr3	45671113	45674887	chr3:45671113-45674887	LIMD1	protein_coding
chr5	107685689	107690120	chr5:107685689-107690120	FBXL17	protein_coding
chr5	158420317	158428595	chr5:158420317-158428595	EBF1	protein_coding
chr5	171472953	171473127	chr5:171472953-171473127	STK10	protein_coding
chr10	6740203	6740440	chr10:6740203-6740440		
chr12	14146304	14146602	chr12:14146304-14146602		
chr14	21957914	21958338	chr14:21957914-21958338	TOX4	protein_coding
chr17	20505874	20506315	chr17:20505874-20506315		
chr6	20139841	20140327	chr6:20139841-20140327	MBOAT1	protein_coding
chr1	25003487	25004000	chr1:25003487-25004000		
chr16	68717631	68718146	chr16:68717631-68718146	CDH3	protein_coding
chr20	62498144	62498669	chr20:62498144-62498669	TPD52L2	protein_coding
chr3	126067331	126067960	chr3:126067331-126067960	KLF15	protein_coding
chr17	44097239	44097903	chr17:44097239-44097903	MAPT	protein_coding
chr20	55195545	55196485	chr20:55195545-55196485		
chr21	45684989	45686004	chr21:45684989-45686004		
chr8	144024072	144029344	chr8:144024072-144029344		
chr12	14639247	14646989	chr12:14639247-14646989	ATF7IP	protein_coding
chr16	66897412	66897556	chr16:66897412-66897556	NAE1	protein_coding

chr4	86266752	86266912	chr4:86266752-86266912		
chr16	13584445	13584625	chr16:13584445-13584625		
chr15	80433857	80434072	chr15:80433857-80434072		
chr15	47709986	47710225	chr15:47709986-47710225	SEMA6D	protein_coding
chr20	1457085	1457328	chr20:1457085-1457328	SIRPB2	protein_coding
chr2	88620768	88621023	chr2:88620768-88621023		
chr20	587391	587654	chr20:587391-587654	TCF15	protein_coding
chr14	50606162	50606431	chr14:50606162-50606431	SOS2	protein_coding
chr3	61721561	61721835	chr3:61721561-61721835	PTPRG	protein_coding
chr11	64596261	64596539	chr11:64596261-64596539	CDC42BPG	protein_coding
chr19	50841547	50841825	chr19:50841547-50841825	NR1H2	protein_coding
chr2	242594438	242594729	chr2:242594438-242594729	ATG4B	protein_coding
chr19	40320033	40320342	chr19:40320033-40320342	DYRK1B	protein_coding
chr11	72830300	72830628	chr11:72830300-72830628	FCHSD2	protein_coding
chrX	13530445	13530781	chrX:13530445-13530781		
chr16	69185271	69185609	chr16:69185271-69185609	CIRH1A	protein_coding
chr1	221555495	221555840	chr1:221555495-221555840		
chr15	86863084	86863446	chr15:86863084-86863446	AGBL1	protein_coding
chr9	2391467	2391835	chr9:2391467-2391835		
chr1	249112984	249113377	chr1:249112984-249113377	SH3BP5L	protein_coding
chr19	555145	555541	chr19:555145-555541		
chr16	82367582	82367983	chr16:82367582-82367983		
chr11	118891741	118892162	chr11:118891741-118892162	TRAPPC4	protein_coding
chr1	217803928	217804353	chr1:217803928-217804353	GPATCH2	protein_coding
chr11	117445644	117446092	chr11:117445644-117446092	DSCAML1	protein_coding
chr1	154305621	154306077	chr1:154305621-15430607	ATP8B2	protein_codin

			7		g
chr16	89728726	89729202	chr16:89728726-89729202	SPATA33	protein_coding
chr13	26819253	26819738	chr13:26819253-26819738		
chr1	31521856	31522344	chr1:31521856-31522344	PUM1	protein_coding
chr11	132888365	132888854	chr11:132888365-132888854	OPCML	protein_coding
chr22	39398892	39399395	chr22:39398892-39399395		
chr1	17947598	17948125	chr1:17947598-17948125	ARHGEF10L	protein_coding
chr4	55927979	55928531	chr4:55927979-55928531		
chr20	48523505	48524092	chr20:48523505-48524092	SPATA2	protein_coding
chr9	71291881	71292468	chr9:71291881-71292468		
chr11	81561888	81562479	chr11:81561888-81562479		
chr11	17467353	17467956	chr11:17467353-17467956	ABCC8	protein_coding
chr8	10689479	10690083	chr8:10689479-10690083	PINX1	protein_coding
chr1	113327637	113328289	chr1:113327637-113328289		
chr17	7582193	7582848	chr17:7582193-7582848	TP53	protein_coding
chr2	27429872	27430527	chr2:27429872-27430527	SLC5A6	protein_coding
chr10	93569015	93569677	chr10:93569015-93569677	TNKS2	protein_coding
chr1	161675842	161676521	chr1:161675842-161676521		
chr22	32401882	32402564	chr22:32401882-32402564		
chr1	237931015	237931727	chr1:237931015-237931727	RYR2	protein_coding
chr2	233944005	233944719	chr2:233944005-233944719	INPP5D	protein_coding
chr2	45949252	45949969	chr2:45949252-45949969	PRKCE	protein_coding
chr16	89228889	89229615	chr16:89228889-89229615		
chr3	15871941	15872673	chr3:15871941-15872673	ANKRD28	protein_coding
chr12	52677315	52678163	chr12:52677315-52678163	KRT86	protein_coding
chr4	153493871	153494781	chr4:153493871-15349478		

			1		
chr17	32609920	32610859	chr17:32609920-32610859		
chr17	29106536	29107535	chr17:29106536-29107535	CRLF3	protein_coding
chr7	30155416	30156447	chr7:30155416-30156447	PLEKHA8	protein_coding
chr11	11577280	11578419	chr11:11577280-11578419	GALNT18	protein_coding
chr21	46114280	46115430	chr21:46114280-46115430	TSPEAR	protein_coding
chr12	9562710	9563909	chr12:9562710-9563909		
chr6	91093892	91095113	chr6:91093892-91095113		
chr2	183181230	183182465	chr2:183181230-183182465	PDE1A	protein_coding
chr1	118364895	118366303	chr1:118364895-118366303		
chr1	179816768	179818291	chr1:179816768-179818291	TOR1AIP2	protein_coding
chr10	77584319	77585951	chr10:77584319-77585951	C10orf11	protein_coding
chr15	94488945	94490652	chr15:94488945-94490652		
chr3	32440750	32442526	chr3:32440750-32442526	CMTM7	protein_coding
chr12	21732680	21734481	chr12:21732680-21734481	GYS2	protein_coding
chr7	10459170	10461000	chr7:10459170-10461000		
chr10	61851887	61854045	chr10:61851887-61854045	ANK3	protein_coding
chr4	110633663	110635825	chr4:110633663-110635825	PLA2G12A	protein_coding
chr12	22881188	22883505	chr12:22881188-22883505		
chr7	40457590	40460115	chr7:40457590-40460115	SUGCT	protein_coding
chr22	35959189	35961772	chr22:35959189-35961772		
chr3	175710068	175712676	chr3:175710068-175712676		
chr9	135484203	135486985	chr9:135484203-135486985	DDX31	protein_coding
chr8	109595844	109598629	chr8:109595844-109598629		
chr12	89846742	89849597	chr12:89846742-89849597	POC1B	protein_coding
chr20	56338396	56341361	chr20:56338396-56341361		

chr6	20171530	20174527	chr6:20171530-20174527	MBOAT1	protein_coding
chr5	144431287	144434342	chr5:144431287-144434342		
chr5	9193884	9197135	chr5:9193884-9197135	SEMA5A	protein_coding
chr17	17678533	17681917	chr17:17678533-17681917	RAI1	protein_coding
chr2	74542408	74545873	chr2:74542408-74545873	RP11-287D1.3	protein_coding
chr1	26961866	26965666	chr1:26961866-26965666		
chr5	142233283	142237624	chr5:142233283-142237624	ARHGAP26	protein_coding
chr2	114750245	114755016	chr2:114750245-114755016		
chr9	87983707	87989207	chr9:87983707-87989207		
chr5	80749830	80755590	chr5:80749830-80755590	SSBP2	protein_coding
chr7	83783652	83789717	chr7:83783652-83789717	SEMA3A	protein_coding
chr20	17775227	17781533	chr20:17775227-17781533		
chr11	9599597	9606321	chr11:9599597-9606321	WEE1	protein_coding
chr14	48672385	48679630	chr14:48672385-48679630		
chr5	88693485	88701311	chr5:88693485-88701311		
chr10	35826128	35834994	chr10:35826128-35834994	CCNY	protein_coding
chr19	1264057	1264492	chr19:1264057-1264492	CIRBP	protein_coding
chr14	81784423	81784965	chr14:81784423-81784965	STON2	protein_coding
chr22	21204635	21204931	chr22:21204635-21204931	PI4KA	protein_coding
chr1	199814951	199815269	chr1:199814951-199815269		
chr5	159581538	159582002	chr5:159581538-159582002		
chr17	27683411	27683593	chr17:27683411-27683593		
chr17	61595354	61595566	chr17:61595354-61595566	ACE	protein_coding
chr5	117267640	117267879	chr5:117267640-117267879		
chr19	6166963	6167206	chr19:6166963-6167206	RFX2	protein_codin

					g
chr5	122540874	122541179	chr5:122540874-122541179		
chr19	45792075	45792392	chr19:45792075-45792392	MARK4	protein_coding
chr2	106566693	106567013	chr2:106566693-106567013		
chr9	132235136	132235462	chr9:132235136-132235462		
chr8	50290834	50291214	chr8:50290834-50291214		
chr19	13382516	13382898	chr19:13382516-13382898	CACNA1A	protein_coding
chr5	173063324	173063716	chr5:173063324-173063716		
chr1	235428789	235429233	chr1:235428789-235429233	ARID4B	protein_coding
chr1	207392304	207392782	chr1:207392304-207392782		
chr12	115071637	115072210	chr12:115071637-115072210		
chr3	51102356	51102933	chr3:51102356-51102933	DOCK3	protein_coding
chr7	35413084	35413668	chr7:35413084-35413668		
chrX	68464643	68465233	chrX:68464643-68465233		
chr17	1343375	1343988	chr17:1343375-1343988	CRK	protein_coding
chr11	59690997	59691627	chr11:59690997-59691627		
chr20	11530675	11531315	chr20:11530675-11531315		
chr19	16616543	16617262	chr19:16616543-16617262	C19orf44	protein_coding
chr3	195447588	195448355	chr3:195447588-195448355	MUC20	protein_coding
chr4	25584533	25585318	chr4:25584533-25585318		
chr19	39054421	39055259	chr19:39054421-39055259	RYR1	protein_coding
chr16	86593543	86594423	chr16:86593543-86594423		
chr3	78438217	78439408	chr3:78438217-78439408		
chr4	91016396	91017802	chr4:91016396-91017802		
chr14	21328665	21330163	chr14:21328665-21330163		
chr1	74536061	74537706	chr1:74536061-74537706	LRRIQ3	protein_coding
chr20	60616605	60618338	chr20:60616605-60618338	TAF4	protein_coding

chr9	125143093	125144911	chr9:125143093-125144911	PTGS1	protein_coding
chr7	68590465	68592328	chr7:68590465-68592328		
chr9	37975714	37977879	chr9:37975714-37977879	SHB	protein_coding
chr4	69235843	69239230	chr4:69235843-69239230		
chr17	62578130	62582211	chr17:62578130-62582211	SMURF2	protein_coding
chr3	151383140	151387389	chr3:151383140-151387389		
chr20	3376702	3381110	chr20:3376702-3381110	C20orf194	protein_coding
chr1	55517475	55522217	chr1:55517475-55522217	PCSK9	protein_coding
chr5	23879194	23884832	chr5:23879194-23884832		
chr1	187174739	187182571	chr1:187174739-187182571		
chr4	118913974	118922764	chr4:118913974-118922764		
