# Supplementary material for: PAFAH1B3 Exists in Linear Chromosomal and Extrachromosomal Circular DNA and Promotes HCC Progression via EMT
Source: Int J Mol Sci. 2025 Sep 10;26(18):8801. doi: 10.3390/ijms26188801 (PMC12469353; doi:10.3390/ijms26188801)
Supplement: Supplementary file 1 [file ijms-26-08801-s001.zip › Supplementary Table 3.pdf]

Table S3 The basal expression levels of PAFAH1B3 in HepG2 and Huh7 cells.

HepG2:

Sample Name	Target Name	CT	average CT value	Sample Name	Target Name	CT	average CT value
NC-1	PAFAH1B3	23.41	23.32	NC-1	GAPDH	19.12	19.36
NC-1	PAFAH1B3	23.12		NC-1	GAPDH	19.51	
NC-1	PAFAH1B3	23.43		NC-1	GAPDH	19.46	
NC-2	PAFAH1B3	23.28	23.36	NC-2	GAPDH	19.21	19.29
NC-2	PAFAH1B3	23.53		NC-2	GAPDH	19.3	
NC-2	PAFAH1B3	23.26		NC-2	GAPDH	19.37	
NC-3	PAFAH1B3	23.95	23.51	NC-3	GAPDH	19.14	19.21
NC-3	PAFAH1B3	23.17		NC-3	GAPDH	19.06	
NC-3	PAFAH1B3	23.42		NC-3	GAPDH	19.42	

Huh7:

Sample Name	Target Name	CT	average CT value	Sample Name	Target Name	CT	average CT value
NC-1	PAFAH1B3	28.33	28.10	NC-1	GAPDH	14.66	15.19
NC-1	PAFAH1B3	28.78		NC-1	GAPDH	15.22	
NC-1	PAFAH1B3	27.2		NC-1	GAPDH	15.7	
NC-2	PAFAH1B3	26.39	26.97	NC-2	GAPDH	14.1	14.20
NC-2	PAFAH1B3	26.75		NC-2	GAPDH	14.13	
NC-2	PAFAH1B3	27.77		NC-2	GAPDH	14.38	
NC-3	PAFAH1B3	27.68	27.09	NC-3	GAPDH	14.86	14.87
NC-3	PAFAH1B3	27.15		NC-3	GAPDH	14.42	
NC-3	PAFAH1B3	26.43		NC-3	GAPDH	15.34	
