# Supplementary material for: PAFAH1B3 Exists in Linear Chromosomal and Extrachromosomal Circular DNA and Promotes HCC Progression via EMT
Source: Int J Mol Sci. 2025 Sep 10;26(18):8801. doi: 10.3390/ijms26188801 (PMC12469353; doi:10.3390/ijms26188801)
Supplement: Supplementary file 1 [file ijms-26-08801-s001.zip › Supplementary Table 4.pdf]

Table S4 siRNAs sequence information

Name	Sequence (5'-3')
PAFAH1B3 si-1	sense: CCAACAUCCCAACCCACUUTT
	antisense: AAGUGGGUUGGGAUGUUGGTT
PAFAH1B3 si-2	sense: AGAAGAACCGACAGGUGAATT
	antisense: UUCACCUGUCGGUUCUUCUTT
PAFAH1B3 si-3	sense: CCAUCAGCCAUCAUGACAUTT
	antisense: AUGUCAUGAUGGCUGAUGGTT
PAFAH1B3 si-4	sense: CUGGGCUACACACCUGUUUTT
	antisense: AAACAGGUGUGUAGCCCAGTT
negative control	sense: UUCUCCGAACGUGUCACGUTT
	antisense: ACGUGACACGUUCGGAGAATT
