# Supplementary material for: PAFAH1B3 Exists in Linear Chromosomal and Extrachromosomal Circular DNA and Promotes HCC Progression via EMT
Source: Int J Mol Sci. 2025 Sep 10;26(18):8801. doi: 10.3390/ijms26188801 (PMC12469353; doi:10.3390/ijms26188801)
Supplement: Supplementary file 1 [file ijms-26-08801-s001.zip › Supplementary Table 5.pdf]

Table S5 The clinical information of the five patients of HCC.

Patient ID	age	gender	histological grade	MVI
1	64	male	Moderately differentiated	M0
2	58	male	Moderately differentiated	M0
3	54	male	Moderately differentiated	M1
4	72	male	Moderately differentiated	M0
5	68	male	High to moderately differentiated	M0

MVI：Microvascular Invasion
