# Supplementary material for: PAFAH1B3 Exists in Linear Chromosomal and Extrachromosomal Circular DNA and Promotes HCC Progression via EMT
Source: Int J Mol Sci. 2025 Sep 10;26(18):8801. doi: 10.3390/ijms26188801 (PMC12469353; doi:10.3390/ijms26188801)
Supplement: Supplementary file 1 [file ijms-26-08801-s001.zip › Supplementary Table 6.pdf]

Table S6 The Primers used in the qPCR assay

**Name**	**Primer Sequence (5’-3’)**
PAFAH1B3-F	ACATCCGGCCCAAGATTGTG
PAFAH1B3-R	GGGCTGTCGCTCATTCACC
eccDNA PAFAH1B3-F	CCACGCCCAGCTAATTTTGT
eccDNA PAFAH1B3-R	GTGAGCCAAGATTGCACCAT
pGEX-5X-2 -F	GGGCTGGCAAGCCACGTTTGGTG
pGEX-5X-2 -R	CCGGGAGCTGCATGTGTCAGAGG
E-cadherin -F	GTCCTGGGCAGACTGAATTT
E-cadherin -R	GACCAAGAAATGGATCTGTGG
N-cadherin -F	TGGACCATCACTCGGCTTA
N-cadherin -R	ACACTGGCAAACCTTCACG
Vimentin-F	CGAGGAGAGCAGGATTTCTC
Vimentin-R	GGTATCAACCAGAGGGAGTGA
GAPDH-F	GCACCGTCAAGGCTGAGAAC
GAPDH-R	GCCTTCTCCATGGTGGTGAA
