# Supplementary material for: PAFAH1B3 Exists in Linear Chromosomal and Extrachromosomal Circular DNA and Promotes HCC Progression via EMT
Source: Int J Mol Sci. 2025 Sep 10;26(18):8801. doi: 10.3390/ijms26188801 (PMC12469353; doi:10.3390/ijms26188801)
Supplement: Supplementary file 1 [file ijms-26-08801-s001.zip › Supplementary Table legends.pdf]

Table S1 The upregulated differentially expressed eccDNAs detected in at least three pairs of samples,of which 21 were mapped to protein-coding genes

Table S2 The downregulated differentially expressed eccDNAs were detected in at least three pairs of samples,of which 211 were mapped to protein-coding genes

Table S3 The basal expression levels of PAFAH1B3 in HepG2 and Huh7 cells
Table S4 siRNAs sequence information
Table S5 The clinical information of the five patients of HCC
Table S6 The Primers used in the qPCR assay
