# Supplementary figures and images for: PAFAH1B3 Exists in Linear Chromosomal and Extrachromosomal Circular DNA and Promotes HCC Progression via EMT
Source: Int J Mol Sci. 2025 Sep 10;26(18):8801. doi: 10.3390/ijms26188801 (PMC12469353; doi:10.3390/ijms26188801)

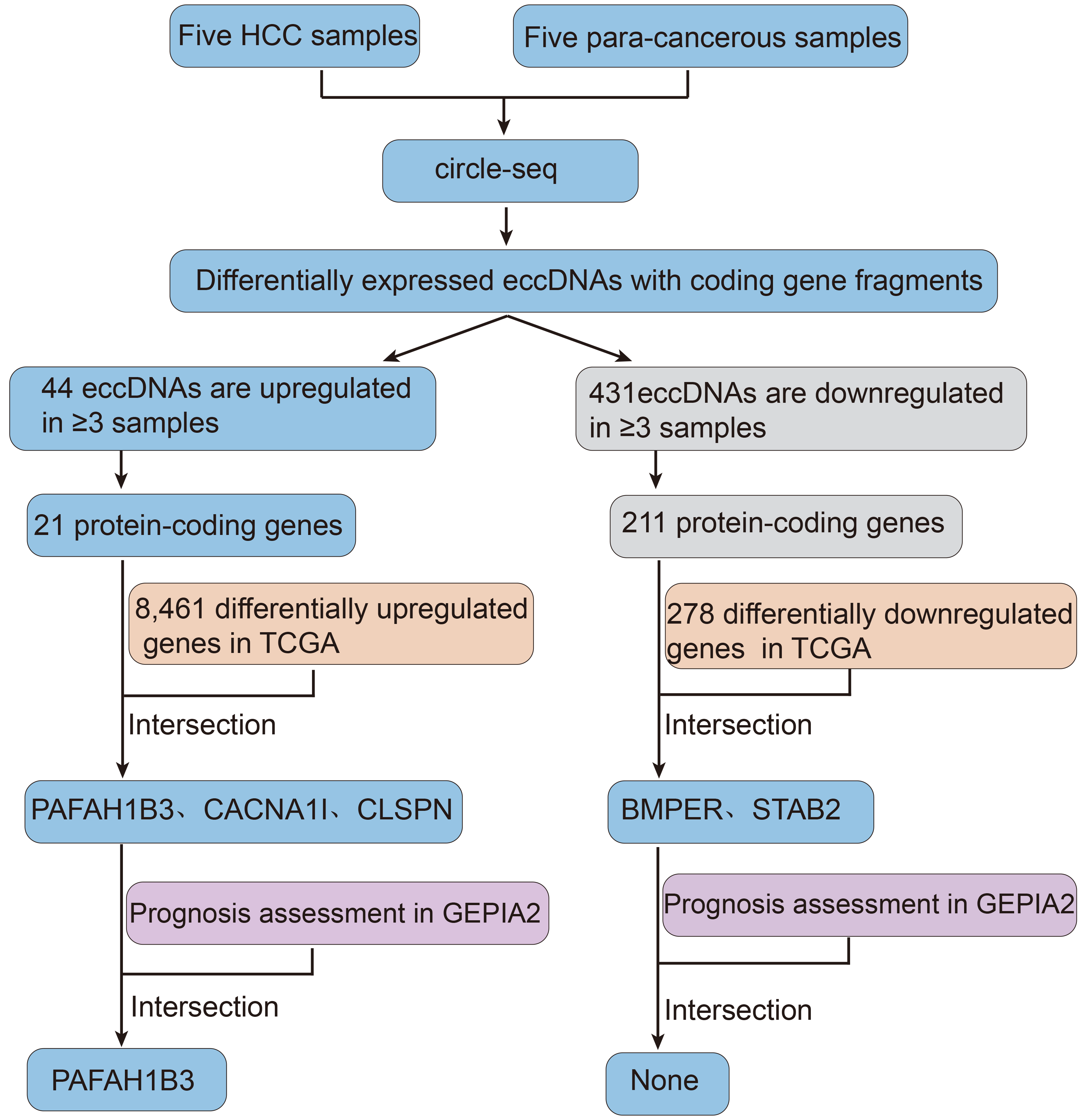

Supplement: Supplementary file 1 [file ijms-26-08801-s001.zip › Fig.S1.tif]

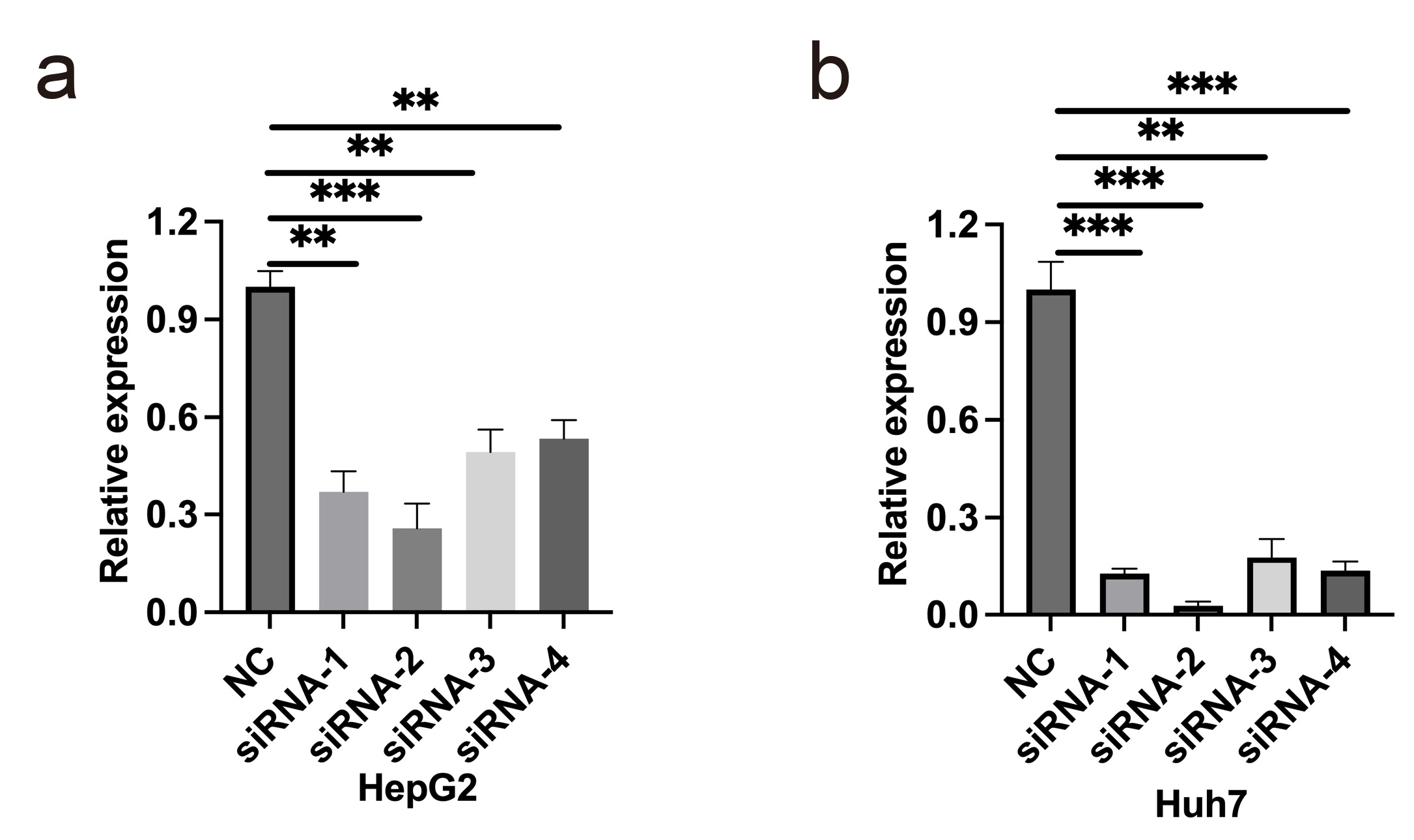

Supplement: Supplementary file 1 [file ijms-26-08801-s001.zip › Fig.S2.tif]

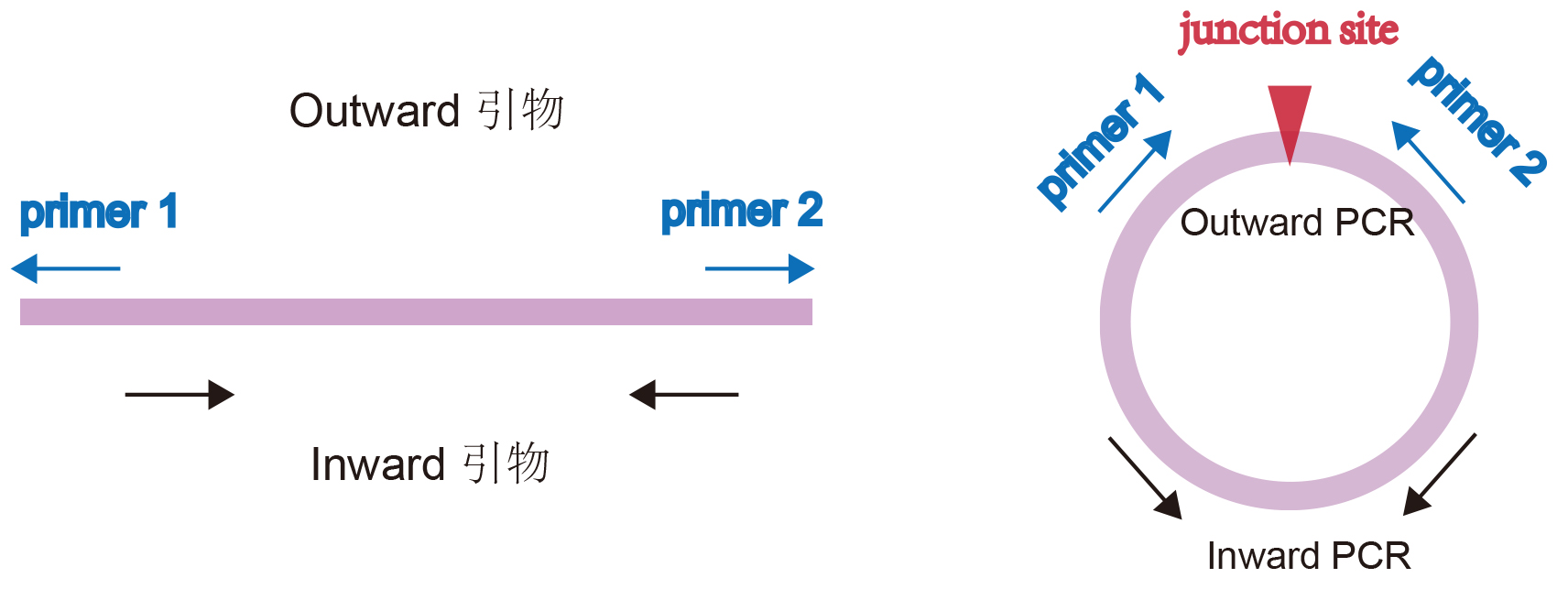

Supplement: Supplementary file 1 [file ijms-26-08801-s001.zip › Fig.S3.jpg]
